# Spotlight on therapeutic efficiency of green synthesis metals and their oxide nanoparticles in periodontitis

**DOI:** 10.1186/s12951-023-02284-5

**Published:** 2024-01-05

**Authors:** Mohammad Kiarashi, Parham Mahamed, Nader Ghotbi, Azadeh Tadayonfard, Kamyar Nasiri, Parisa Kazemi, Ashkan Badkoobeh, Saman Yasamineh, Ali Joudaki

**Affiliations:** 1 College of Dentistry, Lorestan University of Medical Sciences, Khorramabad, Iran; 2 Student Research Committee, Alborz University of Medical Sciences, Karaj, Iran; 3 General Dentist, Isfahan Azad University, School of Dentistry, Isfahan, Iran; 4 Maxillofacial prosthetics fellow, Postgraduate department of prosthodontics, Dental Faculty,Tehran University of Medical Sciences, Tehran, Iran; 5 Department of Dentistry, Islamic Azad University of Medical Sciences, Tehran, Iran; 6 Faculty of Dentistry, Ilam University of Medical Sciences, Ilam, Iran; 7 Department of Oral and Maxillofacial Surgery, School of Dentistry, Qom University of Medical Sciences, Qom, Iran; 8 Azad Researchers, Viro-Biotech, Tehran, Iran; 9 Young Researchers and Elite Club, Tabriz Branch, Islamic Azad University, Tabriz, Iran; 10 Department of Oral and Maxillofacial Surgery, Lorestan University of Medical Sciences, Khorram Abad, Lorestan, Iran

**Keywords:** Green synthesize, Periodontitis, Metal oxide Nanoparticles, Metal nanoparticles, Antibacterial

## Abstract

Periodontitis, one of the most prevalent dental diseases, causes the loss of bone and gum tissue that hold teeth in place. Several bacteria, commonly present in clinically healthy oral cavities, may induce and perpetuate periodontitis when their concentration rises in the gingival sulcus. Antibacterial effect against various Gram-negative and Gram-positive bacteria, including pathogenic and drug-resistant ones, has been shown for several distinct transient metal and metal oxide NPs. Therefore, NPs may be used in biomedicine to treat periodontal problems and in nanotechnology to inhibit the development of microorganisms. Instead of using harmful chemicals or energy-intensive machinery, biosynthesis of metal and metal oxide nanoparticles (NPs) has been suggested. To produce metal and metal oxide NPs, the ideal technique is “Green” synthesis because of its low toxicity and safety for human health and the environment. Gold NPs (AuNPs) appear to be less toxic to mammalian cells than other nanometals because their antibacterial activity is not dependent on reactive oxygen species (ROS). AgNPs also possess chemical stability, catalytic activity, and superior electrical and thermal conductivity, to name a few of their other advantageous characteristics. It was observed that zinc oxide (ZnO) NPs and copper (Cu) NPs exhibited discernible inhibitory effects against gram-positive and gram-negative bacterial strains, respectively. ZnO NPs demonstrated bactericidal activity against the microorganisms responsible for periodontitis. Medications containing magnetic NPs are highly effective against multidrug-resistant bacterial and fungal infections. The titanium dioxide (TiO_2_) NPs are implicated in elevating salivary peroxidase activity in individuals diagnosed with chronic periodontitis. Furthermore, specific metallic NPs have the potential to enhance the antimicrobial efficacy of periodontitis treatments when combined. Therefore, these NPs, as well as their oxide NPs, are only some of the metals and metal oxides that have been synthesized in environmentally friendly ways and shown to have therapeutic benefits against periodontitis.

**Graphical Abstract:**

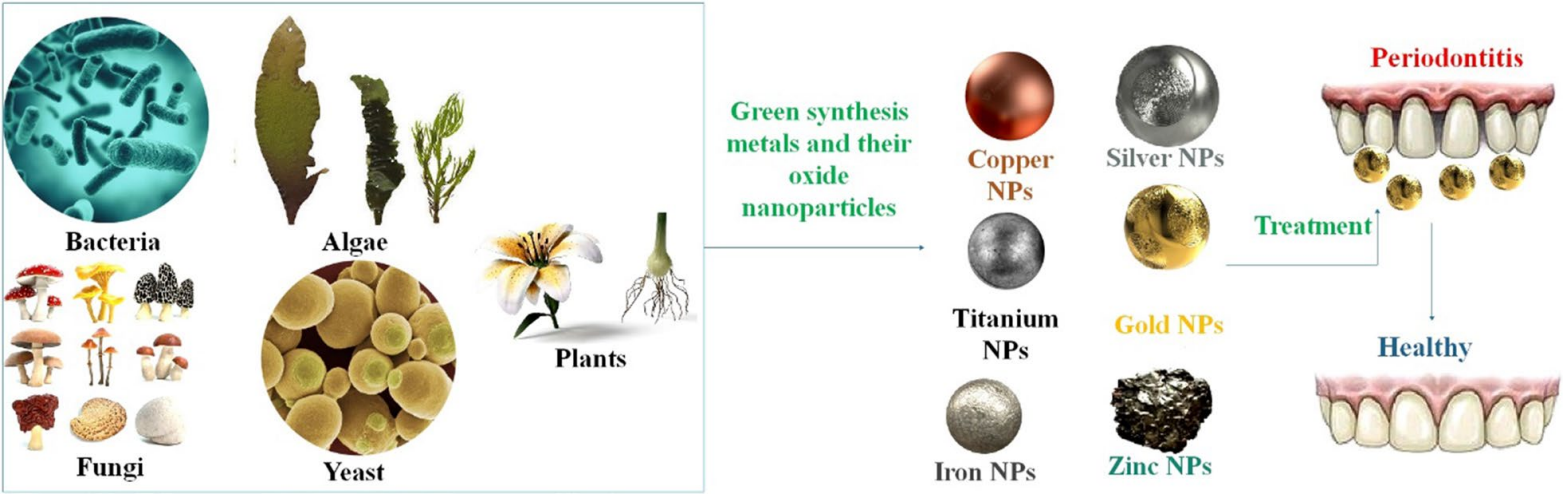

## Introduction

**Fig. 1:**
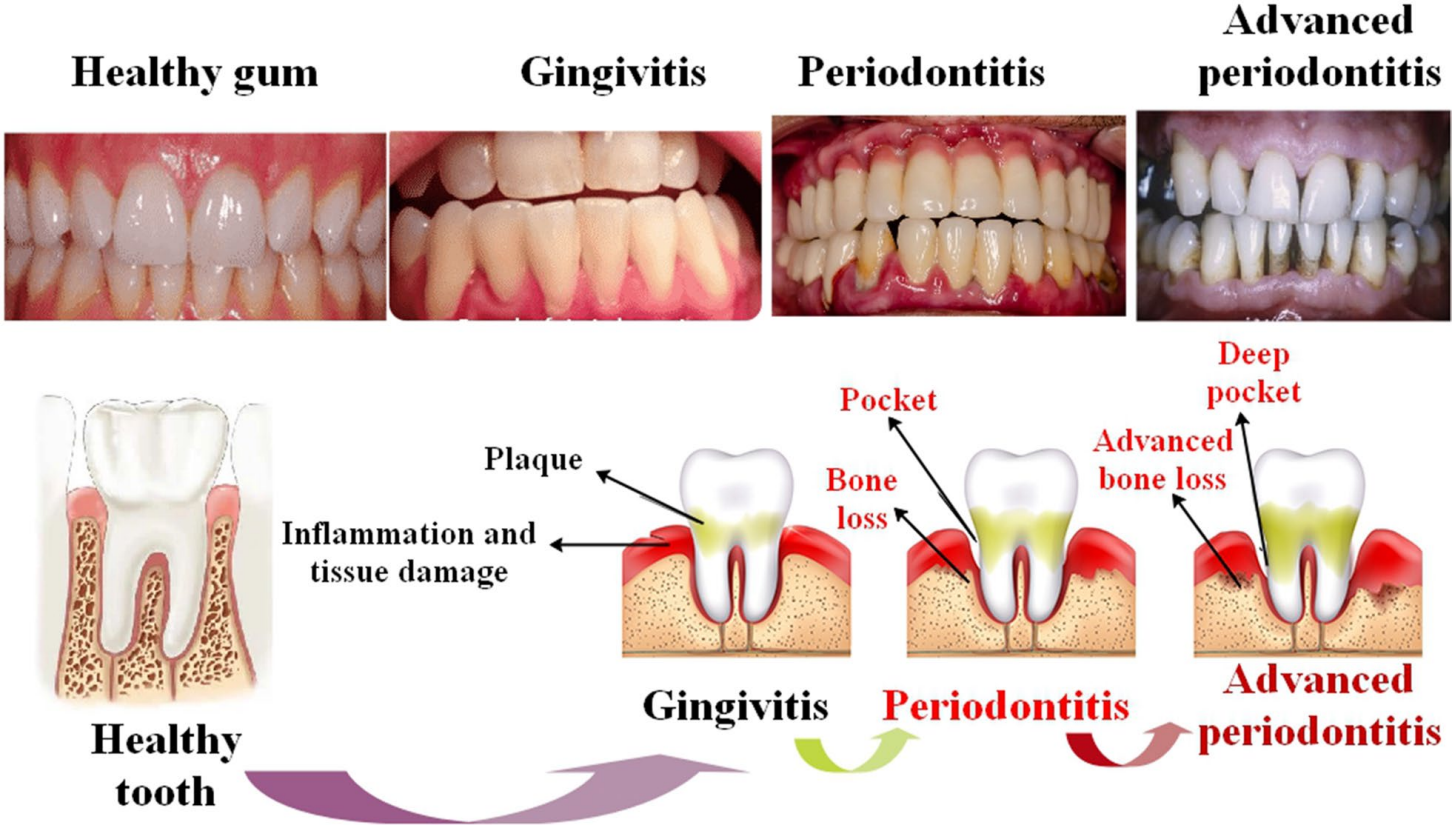
A diagrammatic depiction of periodontitis. Gingivitis is distinguished by the presence of inflamed, red, and oozing gums that encircle the teeth. Although periodontal disease exhibits similar symptoms, it additionally manifests as bone loss. A viscous substance called plaque, which is produced in the oral cavity by food, saliva, and bacteria, irritates the gum tissue by coating the tooth both above and below the gumline. Plaque, if not eliminated, solidifies into calculus, a substance that becomes exceedingly challenging to remove. Plaque and calculus microorganisms have the potential to eventually obliterate the bone and gingival tissue that surround the teeth. This results in the formation of deep fissures, bone atrophy, and potential tooth loss [12]

There is a pressing need for more potent treatments since bacteria resistance to current antibiotics is now recognized as a significant health concern. To stop plaque from forming and maturing, primary prevention via the development of innovative, targeted, and effective antimicrobial medications is essential. Antimicrobial effects on periodontal disease-causing bacteria and the synergistic effects of metal NPs with antibiotics are essential research areas[1]. In many nations, periodontal disease ranks high among top public health concerns [2]. Chronic, inflammatory, and infectious, it manifests as damage to teeth and their supporting tissues, with severe instances resulting in profuse bleeding and inflammation of the gums, loose teeth, and eventual loss. Research suggests that *Pseudomonas aeruginosa* (*P. aeruginosa*), *Escherichia coli* (*E. coli*), *S. pyogenes*, and *B. cereus* may all become active if the microbiota is disturbed. Among chronic patients, *B. cereus*, *S. pyogenes*, *P. aeruginosa*, and *E. coli* tend to be found in disproportionately large numbers at the periodontal site. Recent studies have also pointed to periodontal disease as the most prevalent microbial infection of the mouth [3]. Scaling and root planning are non-surgical therapies for periodontitis that include thoroughly cleaning the teeth and gums to get rid of tartar and germs. In some instances, periodontitis may need surgical intervention for treatment. These might consist of bone and tissue transplants to replace lost bone and tissue, as well as flap surgery, which lifts the gums to get rid of germs and tartar. Antibiotics may be used to lower the amount of germs linked to periodontitis or to stop the tooth's connection to the bone from being destroyed [4]. Gum disease is caused by bacteria, which may be treated with antibiotics. Antibiotics, including tetracyclines, metronidazole, and amoxicillin, are often used for periodontitis. Pain and swelling from periodontitis may be treated with anti-inflammatory medications. These medications usually take the form of nonsteroidal anti-inflammatory medicines (NSAIDs). NSAIDs reduce inflammation and discomfort by preventing the body from making prostaglandins. Ibuprofen and aspirin are two common NSAIDs. Moreover, the use of dual medication delivery has potential for the treatment of periodontal conditions. Illustrative instances include the use of in situ forming gel (ISFG) containing doxycycline hyclate and ibuprofen, as well as the application of in situ forming matrix (ISFM) including vancomycin hydrochloride (VH) and borneol. An alternative methodology is the use of drug-eluting implants, which are inserted directly into the periodontal pocket [5, 6] (Fig. 1).

Determining the cause and timing of periodontitis is the initial obstacle in its treatment. Early disease diagnosis and treatment are crucial in averting subsequent complications. Owing to the absence of discomfort, patients seldom seek medical attention. The second obstacle consists of accurately diagnosing and managing every factor contributing to this illness. Bacteria are the principal causative agents of periodontal diseases, inflicting both direct and indirect harm upon the supporting tissues of the host. Practically all instances of chronic periodontitis can be effectively controlled through scaling and root planing, which involve the mechanical removal and reduction of calculus and bacterial bulk in the subgingival environment. Taking care of the periodontium over time is the third most difficult part of treating chronic periodontitis. Periodontal maintenance, another name for this part of therapy, is what it's called. During this part of therapy, it can be hard to keep the patient motivated and on track, keep an eye on all their risk factors, and then make the right choices about retreatment when needed [7]. Therapeutic intervention encompasses implementing behavior modification strategies, including but not limited to the following: personalized oral hygiene directives, a smoking cessation program, dietary modifications, subgingival instrumentation for calculus and plaque removal, local and systemic pharmacotherapy, and a range of surgical procedures. Supplementary antimicrobial chemotherapy is advantageous for virtually all mechanical periodontal treatments, and no single treatment option has demonstrated superiority. Periodontal treatment necessitates a continuous dedication to meticulous oral hygiene practices due to the chronic nature of the disease. When implemented appropriately, these techniques reduce the likelihood of disease initiation and progression [8]. Cleaning the subgingival infection and getting rid of the periodontal area are also essential parts of treating periodontitis. Although treatment for one to three days seems to be enough to ease the symptoms of periodontal disease, it does not seem to stop the condition from coming back. Some treatments might work best when given through a mix of different methods. A short-acting disintegrating method may be helpful for the first treatment because it can provide a sterilizing dose of the antibacterial agent in the periodontal pocket. Long-term transport of antibacterial agents to the area around the pocket's opening may then stop pocket recolonization from the mouth by stopping marginal plaque [9, 10]. The development of biofilm by oral pathogens presents a significant obstacle in developing antibiotic resistance. To prevent or eliminate microbial biofilms, compounds that inhibit adherence factors and extinguish bioactive quorums can be utilized. Further research is necessary to evaluate the efficacy of quorum suppression strategies in combating periodontal pathogen biofilm formation [11].

**Fig. 2:**
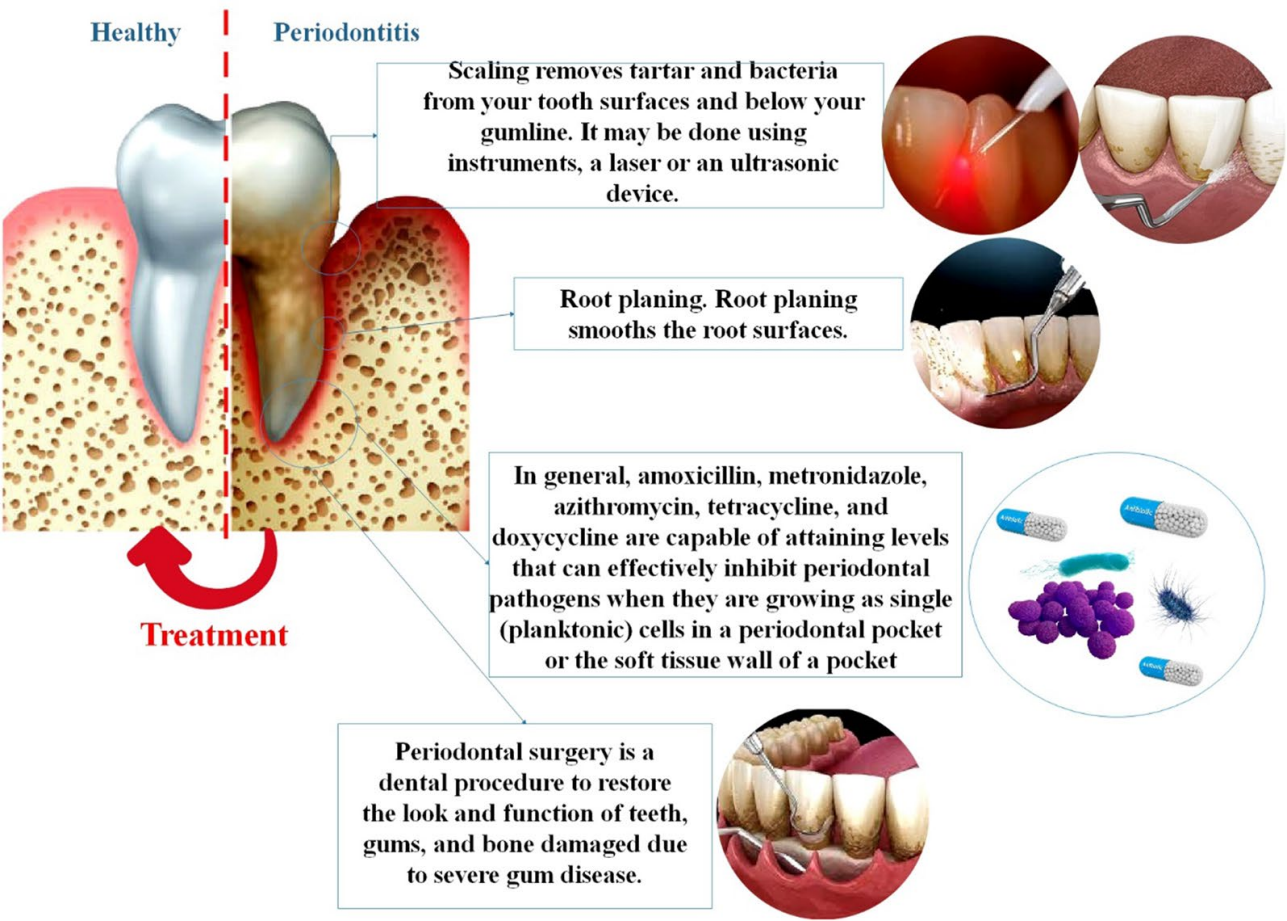
Interventions for periodontitis. The initial line of defense in treating periodontal disease is a thorough cleansing procedure consisting of root planning and debridement. Tartar and pathogens are eliminated from tooth surfaces and below the gingival line through scaling. Utilizing instruments, a laser, or an ultrasonic device, can be achieved. Antibiotics are an additional prevalent non-surgical way to treat periodontal disease. Proficient periodontal antibiotics are specifically formulated for deep pocket placement within the gums by dental practitioners, to address bacterial infections that have spread to the root and gumline. Occasionally, oral antibiotics are required to eliminate infection-causing microorganisms [17, 18]

Antibiotic and anti-inflammatory medications have demonstrated efficacy in enhancing clinical outcomes and impeding the advancement of the disease. Commonly employed systemic antibiotics, such as amoxicillin, metronidazole, and doxycycline, have shown notable effectiveness. However, it is crucial to exercise caution in administering antibiotics, as their indiscriminate use can contribute to the emergence of antibiotic-resistant bacterial strains and other undesirable consequences. The research, testing, and production phases of a new medicine take an average of 13–15 years and cost $2–3 billion in the United States alone. Many prospective medications never make it beyond the first stages of testing in the drug development process, and the success rate is low overall. However, due to rising antibiotic resistance among periodontal pathogens, the focus of periodontitis treatment has switched from killing off bacteria to reestablishing a healthy balance between the oral microbiota and the host periodontal tissue. So, what is required is a systematic medication design that is both modern and effective for the treatment of periodontitis [13] (Fig. 2). Pharmaceutical agents with diverse mechanisms of action may be prescribed to patients harboring antibacterial-resistant microorganisms, among others, due to their susceptibility to such pathogens. Furthermore, these agents may be prescribed singly or in combination to further their utility [14, 15]. After risky periodontal surgeries, patients are often given antibiotics as a preventative measure. But, in line with the current trend of human bacteria becoming more antibiotic-resistant, drug resistance has also grown in people with gum disease over the past few years. Antibiotics don't work as well on these germs because of the particular environment in the gum area and the way biofilm forms. For gum diseases, we need new ways to treat them [11, 16].

Nanotechnology offers a cutting-edge approach to today's pressing challenges. Nanotechnology applications offer a perfect and correct option in dentistry and seem to have answers to the issues that come up with regular dental practices. These brand-new NPs can closely match the surface and contact features of tooth tissues [19]. With the development and implementation of NPs/nanocarriers, nanotechnology has found widespread use across various disciplines. Because of their small size, NPs may easily penetrate the defenses of living organisms. Nanobiomedical knowledge has also been studied for its potential applications in areas including imaging and early-stage illness diagnostics, as well as the efficient and targeted delivery of drugs, genes, and therapeutic compounds to specific organs or cells [20–22]. NPs may be categorized into two distinct kinds, namely organic and inorganic, depending on the composition of their structure [23, 24]. Inorganic NPs are ideal for antigen transport as a vaccination because of their tiny size, greater constancy, controlled adjustability, higher penetrance, superior drug loadings, and activated discharge profile. These productions, known as hybrid inorganic NPs, often have an inorganic core and an organic shell [25–27]. The use of NPs, generally between 0.2 and 100 nm in size, has proven effective as a new antibacterial strategy. Gold (Au), titanium (Ti), silver (Ag), copper (Cu), zinc (Zn), magnesium (Mg), and bismuth (Bi) are the most common metals utilized in biological applications today [28, 29]. Because they may be so tiny, metallic NPs can readily cross the peptidoglycan Accepted Manuscript layer and harm the bacteria inside. Additionally, negatively charged bacterial cell surfaces may absorb Ag^+^, Cu^2+^, and Zn^2+^ ions produced by metallic NPs because of the anionic characteristics of teicoic acids and lipopolysaccharides [30]. Inhibitors of bacterial growth, such as metal NPs, might be effective in treating periodontitis. To treat periodontitis, scientists have developed novel platforms that use metal NPs as effective anti-inflammatory and antibacterial agents [31]. NPs with antimicrobial properties have been identified. These NPs include Au, Ag, Ag_2_O, titanium dioxide (TiO_2_), silicon (Si), Cu oxide (CuO), and zinc oxide (ZnO) [32]. NPs also improve the in vivo effectiveness of bioactive molecules, making them easier to penetrate, improving drug release rates, and allowing for more controlled delivery, all of which are needed for successful periodontal tissue regrowth [33–35]. In conjunction with NPs, recent advances in nanotechnology have made it possible to deliver medications and anti-inflammatory biomolecules to specific sites within the mouth, including periodontal pathogens, inflammatory cells, and periodontal tissues. Highly recommended is additional research evaluating the efficacy of NP-based local drug delivery pharmaceuticals in the treatment of clinical periodontal disease [36]. Periodontal diseases can be found with nanoscale biosensors. With these devices, you can find chemicals in body fluids like spit, blood, and periodontal crevicular fluid [37]. An instance of this is the criticality of multiplex and rapid detection of biomarkers in gingival crevicular fluid (GCF) for the expeditious and accurate diagnosis of the progression and severity of periodontitis. In pursuit of establishing a chairside point-of-care testing (POCT) system for the clinical diagnosis of periodontitis, scientists devised a disk-like lateral flow immunoassay strip (LFIS) employing green core–shell upconversion NPs (G-UCNPs) as the luminescence probe. This strip enables simultaneous detection of three biomarkers associated with periodontitis—namely, interleukin-1 beta (IL-1β), tumor necrosis factor-alpha (TNF-α), interleukin-8 (MPP-8), and interleukin-1 beta (GCF). The three biomarkers were detected with utmost sensitivity and specificity in spiked GCF by the G-UCNPs-LFIS platform, which demonstrated sensitivity limits of 5.455, 0.054, and 4.439 ng/mL, respectively, in standard solutions. Stable and high recovery rates of the three biomarkers were also observed in artificial saliva that had been altered. In addition, the obtained results demonstrated a comparatively high correlation index (0.995 for MMP-8 detection, 0.976 for IL-1β detection, and 0.977 for TNF-α detection) compared to the indices obtained through clinical detection methods. This validates the diagnostic accuracy of the G-UCNPs-LFIS that was developed. Furthermore, the entire detection process was completed in a mere 30 min, allowing users to monitor their periodontitis conditions promptly [38]. To facilitate the early detection of periodontitis, there remains significant demand for sensing materials capable of selectively and accurately differentiating ppb-level methyl mercaptan (CH3SH) from volatile sulfur compounds (VSCs), given that the concentration of CH3SH in exhaled breath marginally increases with the progression of periodontal disease. The principal objective of this study is to develop self-perceived periodontal disease sensors via strategic nanometric lamination of 3 nm-thick AuNPs onto 30 nm-thick ZnO nanofilms. This will be achieved through a two-step procedure involving atomic layer deposition and thermal evaporation. The gas detecting performance was greatly improved when ZnO was coated with AuNPs controlled in size and density. They could respond to 4.99% of the gas for 50 ppb of CH3SH and have a detection limit of 50 ppb. The highly selective detection of ppb-level CH3SH in an H2S atmosphere was possible with Au NP-incorporated ZnO hybrid sensors that had reliable and repeatable gas sensing performance [39].

Physical, chemical, and biological processes may all be used to manufacture NPs. Biological processes are more productive, clean, nontoxic, and ecologically friendly than physical and chemical synthesis, which often includes significant energy consumption and harmful ingredients. Plant extracts, enzymes, and/or microbial synthesis processes have been proposed as more environmentally sound alternatives to conventional chemical synthesis. In response to this international movement, scientists have developed a method called Green Synthesis (GS) that uses plant extracts to create ecological and green NPs. Plants offer several benefits, including being inexpensive to produce, easily accessible, and user-friendly [40]. The productivity of NPs ranging in size, shape, and stability has been increased in different ways recently. Compared to traditional techniques, the yield of metal NPs generated using green approaches is greater, and they are non-toxic. This is because no hazardous by-products are created during the synthesis process [41]. In bio-nanotechnology, the GS of NPs utilizing live cells is an exciting new development. Toxic and hazardous compounds, as well as the inclusion of external reducing, stabilizing, or capping agents, are not used in the GS of NPs, nor is high pressure or temperature necessary [42]. Biological entities, including plant extracts, bacteria, and algae, have been included in the biosynthesis of metal and metal oxide NPs. Compared to algae-, fungi-, and bacteria-based produced NPs, plant-based preparation is a fast, quick, and simple procedure for synthesizing NPs at a large scale. Synthesis of functional nanodevices, production of new medications, and personification of drug delivery processes are only some of the many pharmacological uses for the created green nanomaterials (NMs) [43]. Environmentally friendly "green chemistry" principles have been used in biological production (using organisms like plants, bacteria, fungus, algae, and actinomycetes) of metal or metal oxide NPs [44]. One greener alternative to creating NPs with the appropriate qualities is to synthesize them by living organisms. For biological synthesis, both unicellular and multicellular organisms may respond [45]. Plant-mediated NPs have the benefit of having kinetics that are noticeably greater than those of other biological processes. Due to exceptional phytochemicals, several plant parts, including the leaf, stem, seed, fruit, and roots, have been employed extensively for the production of NPs. To create NPs, certain plant sections are first cleaned with tap or distilled water, then the corresponding salt solutions containing the desired NPs are added, filtered, and squeezed out. The solution's color shifts, indicating the synthesis of NPs, which we can readily separate [46, 47]. In recent decades, the biosynthesis of metal and metal oxide NPs has emerged as a promising study area. To get a procedure that is less harmful to the environment, there has been an increase in the study of green chemistry and the use of green approaches for the synthesis of NPs [47]. Specifically, this article provides a synopsis of green NPs, including Ag, Au, iron, selenium, and Cu, as well as their synthesis, characterization, and potential uses based on plant-based approaches. Emerging biological benefits for green synthesized metal and metal oxide NPs include diagnostics, wound healing, tissue treatment, immunotherapy, regenerative medicine, dentistry (periodontitis), and biosensing platforms [48]. Many oral/dental illnesses may be effectively treated using GS of metal NPs like Ag, Cu, and AuNPs, which are helped by different medicinal plant extracts. Toothpaste and mouthwash, two staples of everyday hygiene, also use these. More study is needed, although there is currently a lack of information on the safety of NMs. Drug resistance is only one problem that might be solved by incorporating a variety of plant extracts into NPs [49]. This article examines the impact of metal NPs and their corresponding oxides and the GS method on the development and progression of periodontitis.

## Biological components for “green” synthesis

Materials science has seen significant interest in the concept of "green" synthesis, which is regarded as a dependable, sustainable, and environmentally friendly approach for producing various materials and NMs. This includes metal and metal oxide NPs, hybrid materials, and bioinspired materials. Therefore, GS is considered a significant approach to mitigate the detrimental impacts associated with conventional synthesizing NPs frequently used in laboratory and industrial settings. To work with various biological materials (such as bacteria, fungi, algae, and plant extracts), GS of metallic NPs has been employed. Compared to synthesis mediated by bacteria and/or fungus, using plant extracts is a very straightforward technique for producing NPs at a large scale, making it one of the more attractive green ways of synthesis for metal/metal oxide NPs. These substances are referred to as biogenic NPs [50]. The use of microbes in the biogenic production of NPs has many benefits over other methods and is being studied more and more [51]. Combining biological extracts with solutions of metal salts allows biogenic synthesis to take place. Variables such as pH, temperature, time, radiation, and metal salt and extract amount are investigated [52]. Biogenic NPs are safe, don’t harm the environment, and help us take a better approach. Biogenic NPs can also be used in many other science and technology areas [53]. Biogenic metallic NPs (BMNPs) are nanostructure materials that have garnered considerable interest owing to their minute dimensions and elevated surface area-to-volume ratio, which are achieved via biological processes [54]. The mechanism by which biogenic metallic or metal oxide NPs kill bacteria often involves the release of metal ions, which interact with cell membranes, cause damage to the cell membranes, and eventually cause the cell membranes to separate into pits or gaps [55]. Biobased GS techniques rely on various reaction parameters, including pH (neutral, basic, or acidic), temperature, pressure, and solvent. Plant biodiversity has been widely taken into consideration for the production of metal/metal oxide NPs because a variety of plant extracts, particularly those from leaves, include potent phytochemicals such as terpenoids, amides, carboxylic acids, flavones, ketones, and ascorbic acids. These elements can turn metal salts into metal NPs [56]. The easiest way and most ecologically friendly procedures may be found in plant extraction techniques, giving them an edge when scaling up for industrial production. Plant chemicals (phytochemicals) may serve a variety of roles, including capping, reducing, and stabilizing agents, in the synthesis processes that lead to the creation of NPs. This GS is an easy, cheap, and fast processing method since it does not involve the use of any potentially dangerous or costly chemicals [57]. The main advantages of using a green method to generate NPs are that it is cheap and simple to implement. However, the tiny size and shape achieved and the unique characteristics of the biological substrates employed mean that green NP production may enhance the characteristics of these NMs [58]. Utilizing a variety of spectroscopic techniques, AgNPs were produced by the synthesis of aqueous root extracts of *Rheum palmatum*. Hexagonal and spherical NPs were discovered. *Staphylococcus aureus* (*S. aureus*) and *P. aeruginosa* were also significantly inhibited by the produced AgNPs, with IC90 values of 15 μg/ml and IC50 values of 7.5 μg/ml, respectively. Bacteria exposed to AgNPs exhibited a significant amount of protein leakage and morphological alterations [59]. Researchers showcase the production of AgNPs utilizing *Massilia* Spp. MAHUQ-52 culture supernatant and the antibacterial use of these NPs against pathogenic *Klebsiella pneumoniae* (*K. pneumoniae*) and *Salmonella Enteritidis*, which are multi-drug resistant. The antibacterial activity of *Massilia* Spp*.* MAHUQ-52 mediated produced AgNPs were determined using the disk diffusion technique against *K. pneumoniae* and *S. Enteritidis*. Biosynthesized AgNPs demonstrated robust antibiotic activity against both *K. pneumoniae* and *S. Enteritidis*. The MICs of produced AgNPs against *K. pneumoniae* and *S. Enteritidis* were 12.5 and 25.0 μg/mL, respectively. The MBC of biosynthesized AgNPs against both bacteria was 50.0 μg/mL. From FE-SEM investigation, it was observed that the AgNPs-treated cells displayed structural alterations with uneven and damaged cell walls that terminated cell death [60]. The past studies involve the production of ZnO-NPs through a fast, cost-effective, and eco-friendly method using four different plant products. The plants used in this study were *Beta vulgaris*, *Cinnamomum tamala*, *Cinnamomum verum*, and *Brassica oleracea var*. *Italica*. All examples of ZnO-NPs showed antibiotic activity against both gram-negative and positive bacteria, while ZnO-NPs made using *Beta vulgaris* was found to be inactive towards *S. aureus*. The antifungal activity of ZnO-NPs was also proven using *Candida albicans* (*C. albicans*) and *Aspergillus niger* (*A. niger*) fungal spots. ZnO-NPs made from *Cinnamomum tamala* were shown to be active against *C. albicans*, whereas those made from *Beta vulgaris* were found to be active against *A. niger*. Additionally, ZnO-NPs made from *Brassica oleracea var*. *italica* extract have shown efficacy against both fungi stains. Overall, it was shown that ZnO-NPs could be synthesized quickly, cheaply, and sustainably. These particles might be used as a possible antibacterial agent against various microbial species [61]. Researchers described a simple method for making MgO NPs using aqueous leaf extracts from *Aloe vera* and *Pisidium guavajava*. The antibacterial activity of produced MgO NPs has been thoroughly investigated against Gram-negative (*E. coli*) and Gram-positive (*S. aureus*) bacteria, which are well-regulated by MgO NPs. Investigators may utilize both precursor materials as a capping and reducing agent (RA). UV absorption shows on 221 nm proves MgNO_3_ reduced as MgO. FTIR results show that plant precursors act as reducing and capping agents. X-ray diffraction shows that the end goods are pure and in face-centered cubic structures (FCC) crystal form. FESEM with EDAX represented the produced MgO NPs having a cubic shape and fully made of MgO. XPS research shows that the as-prepared MgO NPs were made of MgO and plant precursor. Antibacterial action of as-prepared MgO NPs replied to both *E. coli* and *S. aureus* [62].

While GS methods offer innumerable benefits, they also present certain obstacles, such as a scarcity of raw materials and the need to harvest and mature them at specific times, as well as the difficulty of obtaining the desired raw materials [63]. Numerous plant materials can be utilized in the ecological synthesis of NPs; in fact, several researchers have investigated locally obtainable and abundant plants. Although these studies offer the potential for optimizing the utilization of indigenous plants, attaining large-scale worldwide production of GS nanoscale metals remains a formidable challenge. Specific GS processes necessitate exceedingly high temperatures and lengthy synthesis times, which consume substantial amounts of energy and may have negative environmental consequences. Despite utilizing eco-friendly raw materials, the manufacturing process may not entirely adhere to the principles of GS. The properties determined for NPs synthesized by various extracts are inadequate due to the significant variability in size and shape. The primary limitation of this approach is that it generates NPs characterized by defective surface structures. Present reports indicate substantial variations in particle size, rendering green technology unsuitable for large-scale production and posing a formidable challenge in particle size control during production [64, 65].

### Bacterial

Commercial biotechnological applications, such as bioremediation, genetic engineering, and bioleaching, have extensively used bacterial species. Bacteria may decrease metal ions, making them promising candidates for preparing NPs. Many different kinds of bacteria are used to produce metallic and other new NPs. Metal/metal oxide NPs have been widely synthesized using prokaryotic microorganisms and actinomycetes. Since bacteria may be easily manipulated, their production of NPs has become a popular method [66, 67]. Bacterial strains such as *Bacillus cereus* (*B. cereus*), *Bacillus amyloliquefaciens* (*B. amyl*), *Bacillus indicus* (*B. indicus*), and *Bacillus cecembensis* (*B. cecembensis*) have been used extensively in the synthesis of bioreduced AgNPs with varied size/shape morphologies. *Shewanella oneidensis*, *Corynebacterium* Spp. *SH09*, *Aeromonas* Spp. *SH10*, *Phaeocystis antarctica*, *Pseudomonas proteolytica*, *Enterobacter cloacae*, *Geobacter* Spp.., and *Arthrobacter gangotriensis*. Similar to how many different bacteria strains have been employed for the synthesis of AuNPs (including *Bacillus megaterium* D01, *Desulfovibrio desulfuricans*, *E. coli* DH5a, *Bacillus subtilis* (*B. subtilis*) 168, *Shewanella alga*, *Rhodopseudomonas capsulate*, and *Plectonema boryanum* UTEX 485) [50].

### Fungi

Monodispersed NPs with well-defined morphologies may also be efficiently produced by the fungal-mediated biosynthesis of metal/metal oxide NPs. Because they contain many different enzymes inside their cells, they are superior biological agents for making NPs out of metals and metal oxides. More NPs can be synthesized by competent fungi than by bacteria. The abundance of enzymes, proteins, and reducing components on the cell surfaces of fungi is another reason they excel over other species. Metal NPs are thought to arise by enzymatic reduction (reductase) in the fungal cell wall or cytoplasm. Ag, Au, TiO_2_, and ZnO are some of the metals and oxides that may be synthesized using fungi [50]. Using fungi, scientists from around the globe have been able to biosynthesize NPs extracellularly and intracellularly, respectively. Commonly cited examples include *Penicillium* Spp., *Fusarium* Spp., *Fusarium oxysporum*, *Fusarium semitectum*, *Fusarium acuminatum*, *Fusarium solani*, *Cladosporium cladosporioides*, *Trichoderma viride*, and *Aspergillus* Spp. Utilizing fungus, we can compare and contrast the internal and extracellular production of NPs like AgNPs [68].

### Algae

Microalgae, often known as algae, are microorganisms in aquatic environments that perform photosynthesis. It has been shown that algae, like other microorganisms, play a crucial role in the biological production of NMs and the buildup of other heavy metals. On occasion, algae are used in producing ZnO NPs and the industrial production of AU and AgNPs. Microalgae are well-known for their ability to transform potentially toxic metals into their nontoxic analogs. *Phaeodactylum tricornutum* is a kind of microalgae, and its supernatant has recently been employed in the biosynthesis of Ti NPs with a mean particle diameter of 49.7 nm. Because of their cytotoxic, antibacterial, antistatic, and biogenic activities, the NPs generated from microalgae had the potential for use in various biomedical applications. These included imaging methods, hyperthermia, biosensors, drug delivery systems, cancer therapies, and studies of the immune system [69]. In addition, microalgae of the *Sargassum muticum* and *Sargassum myriocystum* were employed to produce 36 nm-sized ZnO NPs. Shape- and size-variable NPs containing carbonyl and hydroxyl groups were created by *S. myriocystum*. In addition, the *S. muticum*-produced NPs were discovered to be hexagonal, and their polysaccharides included both hydroxyl and sulfate groups. Two approaches, including cyanobacterial and microalgal strains were used to evaluate the production of AgNPs, as described in the literature [70, 71]. One technique included adding Ag nitrate (AgNO_3_) to cell-free media, while another involved washing and suspending live biomass from the two groups of bacteria in an AgNO_3_ solution. Using any of these two techniques, fourteen of the sixteen tested strains were able to successfully produce AgNPs with diameters ranging from 13.0–31.0 nm, demonstrating that extracellular components were involved in the creation of AgNPs. Antibacterial activity has also been shown for the AgNPs, except for the largest NPs generated by the *cyanobacterium strain* (*Limnothrix* Spp. 37–2-1). Furthermore, Ag and AuNPs were synthesized using *Chlorella vulgaris*, a nanofactory microalgae. Antibacterial activity was shown for both NPs against *S. aureus*, *Streptococcus* Spp., and *E. coli*. Similarly, tin oxide (SnO_2_) NPs with photocatalytic and biological properties were synthesized greenly using *Chlorella vulgaris*. Antibacterial activity against four pathogenic bacteria, antioxidant properties, cytotoxicity against lung cancer, and photocatalytic breakdown of the methyl orange dye UV light were all shown by the produced NPs. *Padina* Spp. marine macroalgae were utilized to prove that marine macroalgae may be used to synthesize AgNPs. It was discovered that when this particular type of algae was included, the synthesis of AgNPs increased. *S. aureus* and *P. aeruginosa* were both killed by the NPs with inhibition zones of 15 and 13 mm, respectively [68, 72, 73].

### Yeast

Multiple studies have shown the viability of using yeasts to synthesize NMs; yeasts are single-celled eukaryotes that developed from multicellular ones. Many of the roughly 1500 varieties of yeast are widely used to fabricate metallic NMs. Yeasts have large surface areas, making them particularly susceptible to the accumulation of toxic metals [74]. Many laboratories have shown that yeast can successfully synthesize NPs/ NMs. One may use an Ag-tolerant yeast strain and *Saccharomyces cerevisiae* (*S. cerevisiae*) broth to biosynthesize Ag and AuNPs, as was recently described. Numerous metallic NPs are produced using various species [50, 75].

### Plant cell

Plants may store varying levels of heavy metals in their many tissues. Therefore, biosynthetic strategies using plant extracts have received more attention as a straightforward, productive, reasonably priced, and practically doable alternative to standard preparation processes for NP generation [76]. Among the biomolecules found in plants are those with exceptional ability to catalyze the reduction of metal salt into NPs. Plant extract-assisted synthesis was the first biosynthetic route to explore for Au and AgNPs. Various plants, including aloe vera (*Aloe barbadensis Miller*), Oat (*Avena sativa*), alfalfa (*Medicago sativa*), Tulsi (*Osimum sanctum*), Lemon (*Citrus limon*), Neem (*Azadirachta indica*), Coriander (*Coriandrum sativum*), Mustard (*Brassica juncea*) and lemon grass (*Cymbopogon flexuosus*), have been utilized to synthesize AgNPs and AuNPs. Other plants, including mustard (*Brassica juncea*), alfalfa (*Medicago sativa*), and sunflower (*Helianthus annuus*), were shown to produce NPs in vivo as well. Coriander (Coriandrum sativum), crown flower (*Calotropis gigantean*), Cu leaf (*Acalypha indica*), China rose (*Hibiscus rosa-sinensis*), Green Tea (*Camellia sinensis*), and aloe leaf broth extract (*Aloe barbadensis Miller*) are just some of the many plant leaf extracts that have been used to prepare ZnO NPs [46, 77–80].

## Mechanism of “green” synthesis for metals and their oxide nanoparticles

GS has been used by several groups to create metal/metal oxide NPs. Mechanisms for “green” production of metals and their oxide NPs, such as those based on microorganisms and plant leaf extracts, are now under investigation. Furthermore, enzymatic reduction (reductase) in the cell wall or inside the fungal cell is likely the process for creating metallic NPs. Metal/metal oxide NPs, including Ag, Au, TiO_2_, and ZnO, are synthesized using various fungal species [50].

### Microorganism-based mechanism

**Fig. 3:**
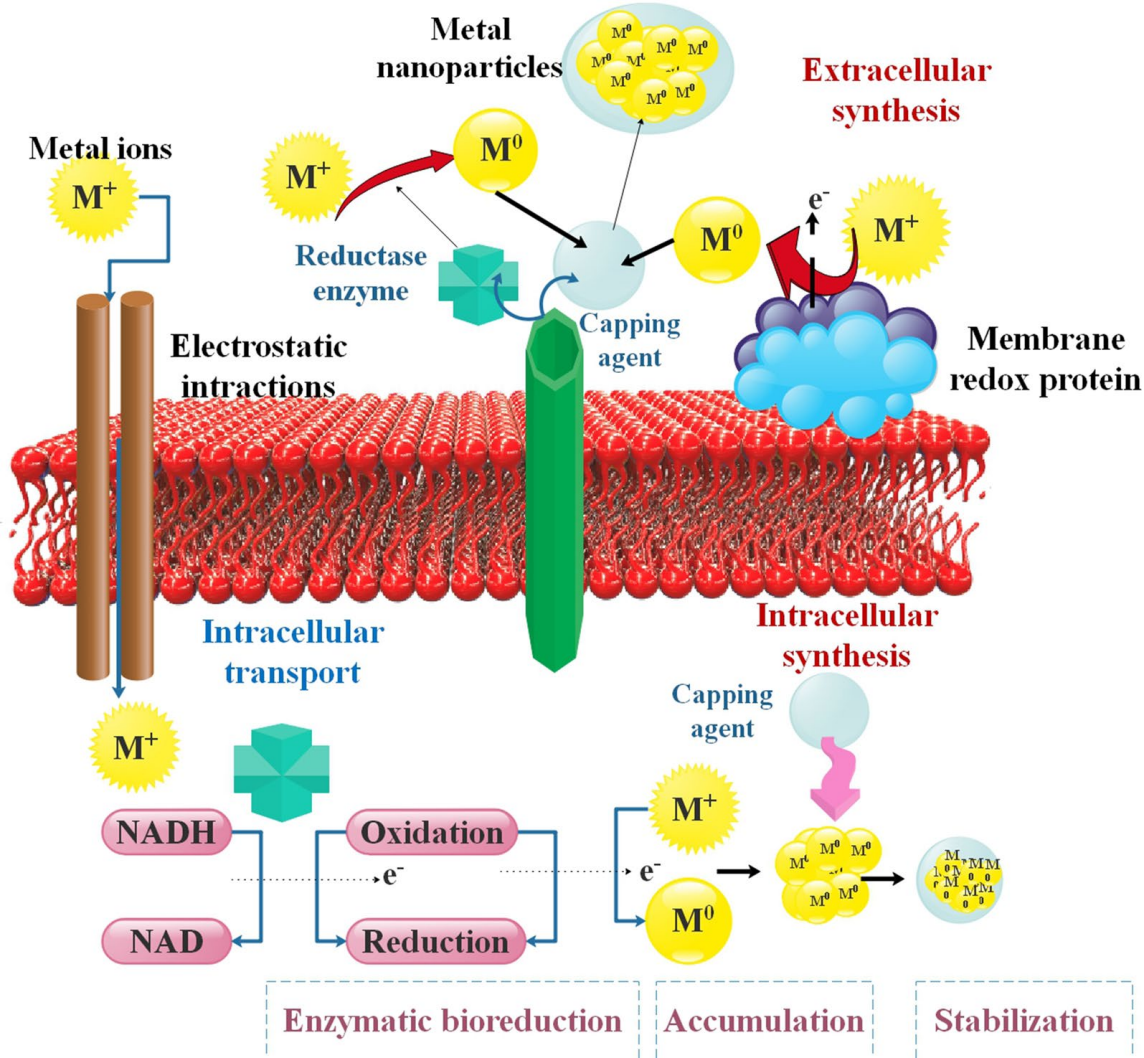
Diagram illustrating the processes involved in the extracellular and intracellular production of metal NPs. metal NPs are produced extracellularly by ensnaring metal ions on the cell wall and reducing them in the presence of metabolites or released enzymes. Following the passage of metal ions into the cytoplasm of the cell, the metal ions are reduced during the intracellular production of metal NPs due to metabolic interactions with enzymes such as nitrate reductase [65]

Different bacteria may use various ways to create NPs. First, metal ions are detained on or inside the microbial cells, and then, thanks to the work of enzymes, they are transformed into metal NPs. Based on the following hypothesis, Sneha et al. [81] outlined the process by which microorganisms aid in forming Ag and AuNPs through *Verticillium* Spp. or algal biomass. Electrostatic interactions between Ag or Au ions and negatively charged cell wall enzymes were the first step in capturing the ions on the surface of fungal cells. Afterward, the Ag or Au nuclei were reduced from ions to produce growth. Both nicotinamide adenine dinucleotide (NADH) and nitrate reductase, which is reliant on NADH, are essential for the production of NPs. The nitrate reductase was shown to be responsible for *B. licheniformis*' bioreduction of AgNPs by Kalishwaralal et al. [82]. Although microorganisms produce metal salt ions and metallic NPs, the bioreduction mechanisms involved in these processes are not well understood [81, 82] (Fig. 3).

### Plant leaf extract-based mechanism

**Fig. 4:**
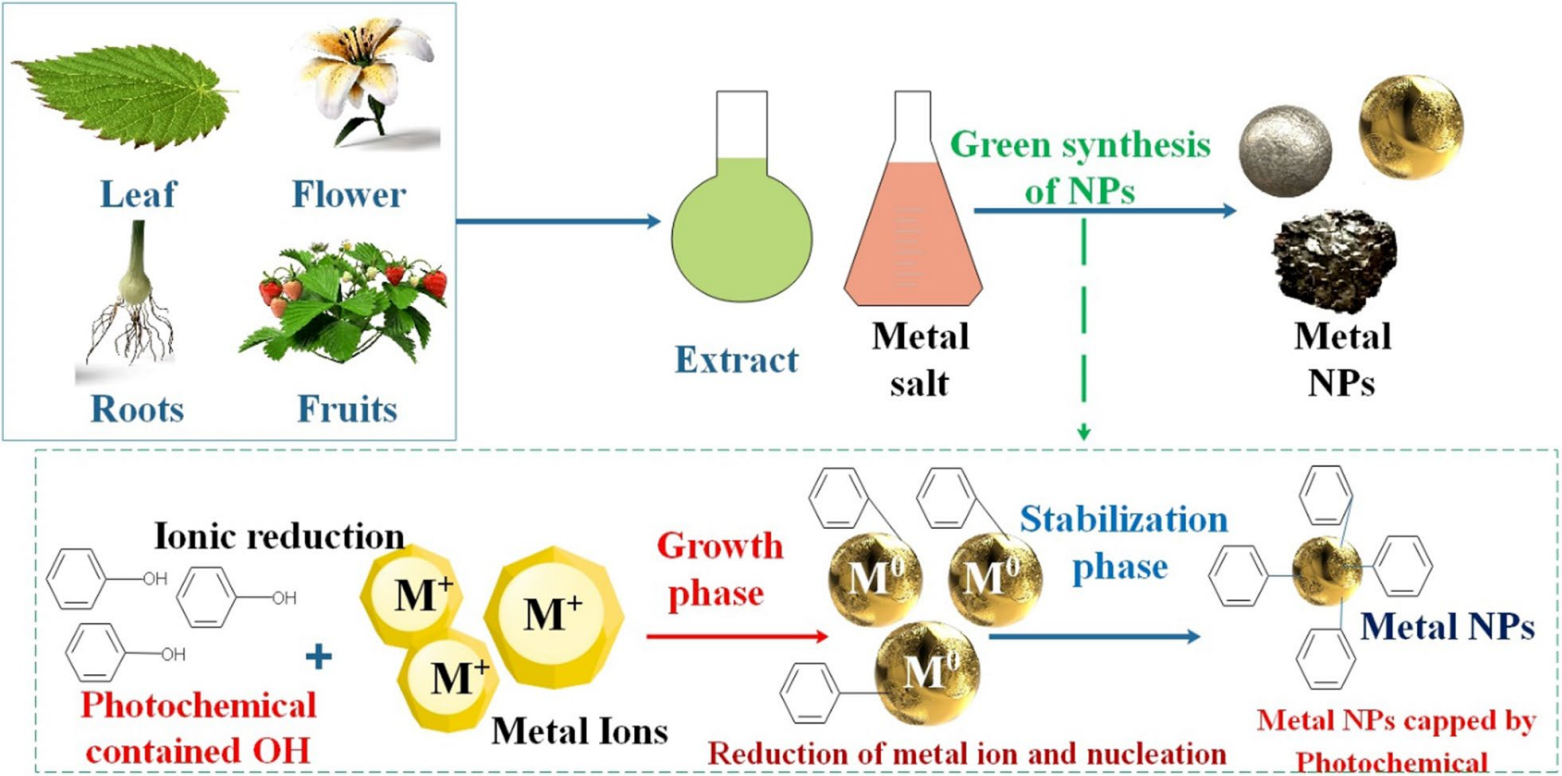
Schematic representation of the mechanisms of green synthesis (GS) of metal and metal oxide NPs by different parts of plants

Plant leaf extract is used as a catalyst in NP production by combining metal precursor solutions under varying reaction conditions. The pace of NP creation, as well as their yield and stability, are allowed to be controlled by the factors governing the circumstances of the plant leaf extract (such as the kinds of phytochemicals, phytochemical concentration, metal salt concentration, pH, and temperature). Plant leaf extracts include phytochemicals with the remarkable capacity to decrease metal ions in much less time than fungus and bacteria, which need a more extended incubation period. Therefore, plant leaf extracts are a great, safe option for making NPs out of metals and metal oxides. In addition, plant leaf extract facilitates the synthesis of NPs by serving as both an RA and a stabilizing agent throughout the synthesis process. In addition to the plant used, the concentration of phytochemicals in the plant leaf extract plays a significant role in NP production. Plants' primary phytochemicals responsible for the bioreduction of NPs include flavonoids, terpenoids, sugars, ketones, aldehydes, carboxylic acids, and amides [50, 83–85]. In general, there are three primary phases to the mechanism of metal NP synthesis in plants and plant extracts: 1) the activation phase, which is when the reduction of metal ions and nucleation of the reduced metal atoms take place; 2) the growth phase, which is when the small adjacent NPs spontaneously merge into larger particles (direct formation of NPs using heterogeneous nucleation and growth, and further metal ion reduction; a process known as Ostwald ripening), which is coupled with an increase in the thermodynamic stability of NPs; and 3) the process termination phase, which determines the final shape of the NPs [86] (Fig. 4). Plant extracts have the potential to function as both stabilizing and RAs during the NP synthesis process. It is recognized that the properties of the NPs can be influenced by the origin of the plant extract. This is because the concentrations and mixtures of organic RAs vary between extracts. A plant extract-mediated bioreduction generally entails the combination of an aqueous solution containing the metal salt in question with the aqueous extract. Within a few minutes, the reaction typically reaches its conclusion at ambient temperature. Due to the multitude of chemical compounds utilized, the bioreduction process is comparatively intricate [83]. Biocompatible, non-toxic NPs are produced via plant-based GS, rendering them a secure and environmentally sustainable substitute for chemical processes, especially in biomedical implementations [87]. Additionally, plant extracts decrease the time necessary to reduce metal ions. This is because phyto nanofabrication does not necessitate the formation of cell cultures, prolonged incubation periods, or elevated temperatures. The expeditious reduction of metal ions can be attributed to the electron-donating capability of plant constituents (functional groups towards metal ion complexes). Natural plant extracts are primarily considered for NPs synthesis due to their potential ecological benefits associated with GS. The primary use of GS is that it permits the selection of solvents, benign materials for stabilization, and environmentally friendly RAs. Plant extracts contain a diverse array of compounds, including amines, amides, alkaloids, flavonoids, phenols, terpenoids, proteins, and carotenoids. The phytochemical components mentioned above contribute to the reduction and stabilization of metal ions throughout the environmentally friendly NP synthesis process [88–91].

## Metal nanoparticles in periodontitis

In reaction to subgingival biofilm, the human immune system triggers persistent inflammation known as periodontitis. Dental scaling and root planning are examples of traditional therapy modalities aimed at reducing subgingival biofilm, and they are often supported with antibacterial coadjuvant treatments such as antibiotics, antiseptics, and probiotics [41]. Antibiotics, antiseptics, and probiotics have frequently been used in conjunction with traditional periodontal management including mechanical debridement and regular dental hygiene practices in the previous two decades. However, getting these medicinal substances where they need to go remains difficult. Systemic antibiotics are seldom recommended for the treatment of periodontal diseases because of the risks associated with their use and the inability to achieve a high enough concentration at the disease site. In addition, Rapid drop in plasma concentration, gastrointestinal intolerance discomfort, microbiological resistance, and hypersensitivity have all been described, leading to sub-therapeutic doses of antibiotics [31]. Some of the local factors that can make periodontal treatment less compelling are the severity of the disease, the number of furcation involved, the patient's ability to control plaque, plaque-retentive factors (such as faulty fillings and cavities), and facial injuries [7]. By eliminating subgingival pathogens that persist following conventional mechanical periodontal therapy, systemic periodontal antibiotic therapy seeks to support the host's defense system in overcoming the infection and to reinforce mechanical periodontal treatment. The effectiveness of systemic antibiotics in treating periodontal diseases may be determined by the susceptibility of bacteria to antibiotics. Inhibiting the enzyme collagenase enables a limited number of chemotherapeutic agents to decrease collagen and bone degradation. Stable adult periodontitis or gingivitis patients typically respond favorably to mechanical periodontal therapy; antibiotic therapy provides minimal or no additional benefit [92]. It's not clear how to best use systemic antimicrobials to treat both short-term and long-term gum illnesses. That being said, the method seems appealing because periodontal diseases are caused by microbes. On the other hand, there is mixed proof that these agents help with most periodontal diseases, and antimicrobials can also hurt. Some problems with systemic antimicrobials are allergic responses, the risk of getting another infection, toxicity, drug combinations, patient cooperation, and, most importantly, germs becoming resistant to the drug. For most periodontal diseases, mechanical cleaning methods, such as draining pus from acute gum abscesses, should be the first choice. Systemic antimicrobials should only be used with mechanical cleaning methods. They should never be used by people with chronic diseases because they can make abscesses more likely to form. When there is an acute disease that is hard to debride or drain pus from, or when there is local spread or systemic upset, additional systemic antimicrobials may be thought of. When someone has a chronic periodontal disease that starts early or gets worse quickly, or when mechanical treatments have failed, and surgery is not a choice, they should think about adding antimicrobials to their treatment plan. If you don't brush your teeth or smoke tobacco, you shouldn't use antimicrobials [93].

A new generation of efficient local drug delivery systems has emerged due to research into the problem, and these systems can increase the concentration of drug molecules at the site of action while reducing or eliminating their systemic harmful effects [94, 95]. Acidogenic plaque bacteria such as *S. mutans*, *S. sobrinus*, and *Lactobacillus* Spp. are involved in the development of the illness [96]. Periodontal disorders, which may affect both the hard and soft tissues, are the most frequent inflammatory degenerative ailments affecting people. Diseases of the gums and supporting structures of the teeth, caused by plaque particles that form on the hard root surface next to the soft tissues of the supporting periodontium, can be superficial (gingivitis) or systemic (periodontitis), destroying the alveolar bone that anchors the teeth in place and the periodontal ligament. Teeth may become loose or fall out if they experience such attachment loss and the subsequent creation of periodontal pockets. Primary periodontal infections include *Porphyromonas gingivalis*, *Prevotella intermedia*, and *Aggregatibacter actinomycetemcomitans* [96, 97].

The quantum size effect causes metal NPs, which are generated by grinding metal into tiny particles (< 100 nm in diameter), to have a different set of characteristics than bulk metals [98]. Their very small size gives them unique qualities, like better contact with cells, because they have a bigger surface area-to-mass ratio and the ability to be used in various ways that can be controlled [99]. Most of the time, smaller NPs are more effective at killing bacteria because they can get inside cells and stop bacteria from growing. Also, smaller NPs have a higher surface area-to-volume ratio than bigger NPs, which helps ROS generation [100]. Numerous disciplines have investigated metal NPs, promising results emerging in recent years. These disciplines, includeing chemistry, biology, materials science, and medicine, are not new information. AgNPs, in particular, have garnered attention for their potent antimicrobial properties across various disciplines. Since AuNPs are both inert and very stable, they are often investigated for potential use in biosensing or drug delivery applications. Coating AuNPs with organic compounds that exhibit antibacterial characteristics is one method for making antibacterial AuNPs [101].

NPs have been subject to a comprehensive investigation of various biological applications. Metal-based NPs have shown efficacy against priority pathogens, in addition to their decreased size and selectivity for bacteria, as stated by the World Health Organization (WHO) [102]. Not only is it more challenging for bacteria to acquire resistance to metal-based NPs because they do not bind to a particular receptor in the bacterial cell, but the range of their antibacterial action is also expanded. Therefore, most research on the effectiveness of metal-based NPs has shown encouraging results in both Gram-positive and Gram-negative bacteria. Researchers will find that although other NPs often utilized in antibiotherapy (such as Au, ZnO, Cu, and CuO NPs) are also studied, AgNPs get the lion's share of the attention [103]. Inhibitors of bacterial growth, such as metal NPs, might be effective in treating periodontitis. To treat periodontitis, scientists have developed novel platforms that use metal NPs as effective anti-inflammatory and antibacterial agents [104].

**Fig. 5:**
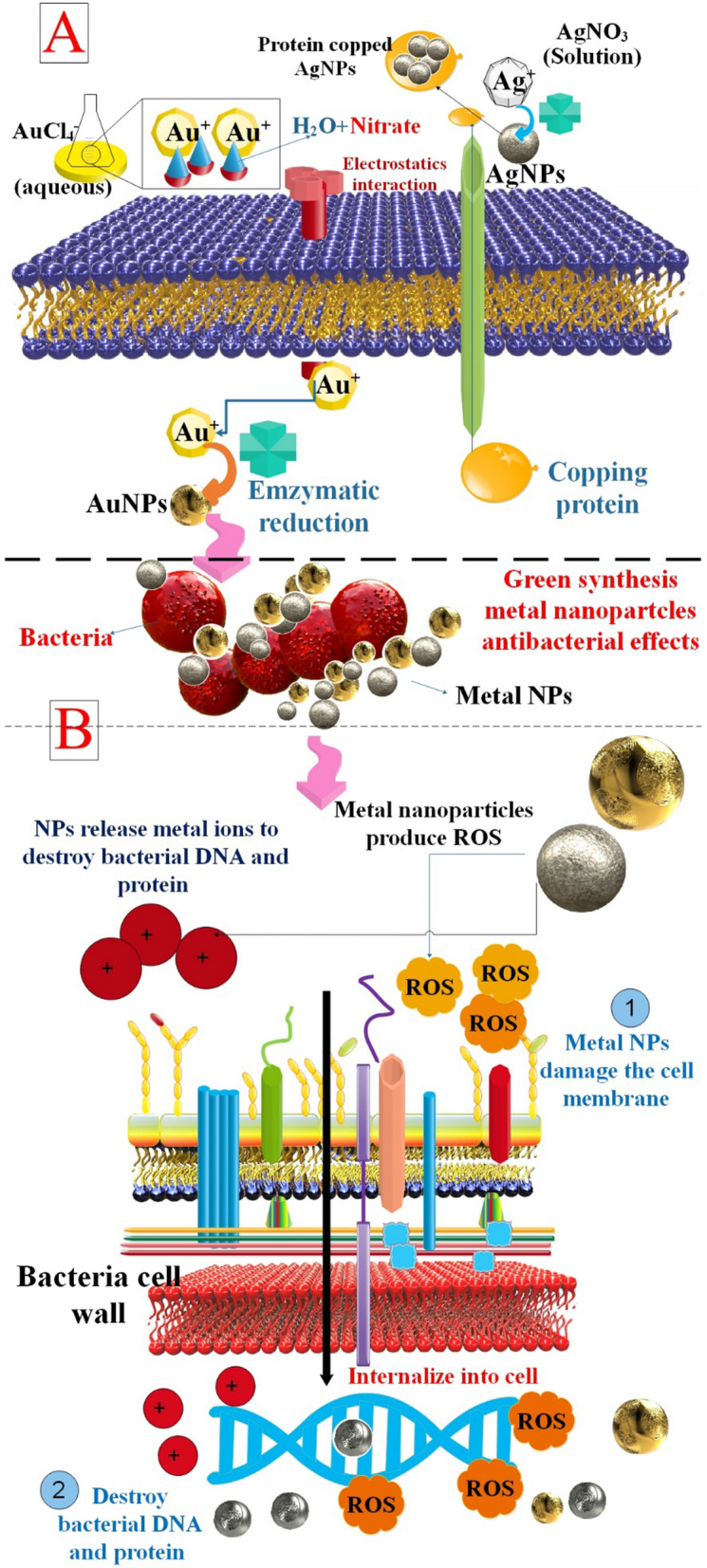
metal and metal oxide NPs such as AgNPs and AuNPs have green synthesis mechanism (**A**) and antibacterial effects (**B**)

Metallic NPs have shown efficacy against *E. coli*, *S. aureus*, *B. subtilis*, and *Salmonella*, serving as a potential substitute for standard antibacterial nanobiotics. Nevertheless, it is widely accepted that the detrimental antibacterial effects of metallic NPs may be attributed to three main mechanisms: the production of reactive oxidative species (ROS), the liberation of ions, and the interaction between NPs and the cellular membrane. Metallic NPs, compared to their corresponding salts, have heightened efficacy in addressing bacterial infections. Primarily, the magnitude of NP dimensions has a significant impact on the antibacterial mechanism [105]. Metal NPs, including Au, Ag, Ag_2_O, TiO_2_, Si, CuO, ZnO, Au, calcium oxide (CaO), and magnesium oxide (MgO), have been shown to have antimicrobial action [106]. These NPs produce ROS, which are hazardous to microorganisms because they may break down cellular components, including DNA, RNA, and proteins. Compared to the other nanometals, AuNPs are less hazardous to mammalian cells because their antibacterial effect does not depend on ROS. As a result of their high functionalization potential, these NPs are also promising candidates for use as targeted antibacterial NMs. ZnO NPs also have great photocatalytic activity, which boosts their antibacterial efficiency. ZnO NPs are also capable of producing ROS when exposed to UV light [107]. In addition, the metal ions given off by these NPs are lethal to the DNA and protein of bacteria. When metal NPs contact bacteria, their negatively charged membranes attract the positively charged metal ions. Bacterial membranes are porous, allowing metal ions within the cells. In doing so, they react with the sulfhydryl group (-SH) on microbial proteins, rendering them incapable of producing proteins or nucleic acids. The ability of NPs to kill germs depends on factors, including their particle size and surface charge. Because of their smaller particle size and better surface-to-volume ratio, they are very effective against germs without compromising the material's mechanical properties. In addition to direct contact with NPs, another method for killing bacteria is by the release of the loaded antibacterial chemical [31, 108] (Table 1) (Fig. 5). To determine whether they may be used to treat periodontitis, researchers created, produced, and characterized AgNPs conjugated with either metronidazole (AgNPs-PEG-MET) or chlorhexidine (AgNPs-CHL). While AgNPs-CHL was more cytotoxic than AgNPs-PEG-MET, it turned out to be a more effective antibacterial agent; both, however, showed positive characteristics at nontoxic doses. AgNPs-CHL and AgNPs-PEG-MET inhibited the production of TNFα, IL-1β, IL-6, and other proinflammatory cytokines. The metalloproteinases MMP3 and MMP8 were likewise reduced by both treatments, which may mean they will prevent tissue deterioration [109]. This study found that 45 nm AuNPs could significantly reduce inflammation and enhance the inflammatory microenvironment of the periodontal ligament by controlling the production of inflammatory and regenerative cytokines and modulating macrophage polarization, which in turn affects the differentiation of hPDLCs (human periodontal ligament cells). Treatment with 45 AuNPs resulted in a significant increase in newly-formed periodontal attachment, bone, and cementum in periodontal defects and reduced tissue destruction as periodontitis progressed. This study showed that 45-nm AuNPs could not only change hPDLCs directly, but they could also change the early inflammatory response of periodontal tissues by controlling the phenotypes of macrophages. This created a microenvironment with low levels of inflammatory cytokines and high levels of reparative cytokines like bone morphogenetic protein-2 (BMP-2), which led to PDLC differentiation, periodontal tissue regeneration, and stopping the progression of periodontitis [110]. Comparing ZnO NPs to other NPs, including Ag, they are more biocompatible. However, because of the metal's intrinsic toxicity and the solubility of the NPs, which is influenced by the metal's chemical makeup, absorption, and capacity to cause oxidative stress, it may be lethal at large concentrations. Conversely, the administration of lesser doses lessens its antibacterial effects. ZnO NPs generate ROS in response to light (365–400 nm) or ultrasonic vibrations, which at low concentrations increases their antibacterial activity and causes oxidative stress. As a result, this study looked at how well ROS-based antimicrobial strategies worked against the polymicrobial periopathogenic biofilms (*P. gingivitis*, *P. intermedia*, and *A. actinomycetemcomitans*) that formed on mini-screws coated with ZnO NPs. It also looked at how these biofilms affected the levels of inflammatory factors (TNF-α, IL-1β, and IL-6) released by human gingival fibroblast (HGF) cells around the mini-screws. The study found that using ZnO NPs in photo-sonodynamic treatment to kill microbes could greatly lower the amount of periopathogen biofilm and inflammatory cytokines in the body [111]. In another study, scientists evaluated whether TiO_2_ NPs activate COX-2 and looked into the molecular pathways that underlie TiO_2_ NPs’ pro-inflammatory effects on human periodontal ligament (PDL) cells. TiO_2_ NP treatment of PDL cells resulted in the upregulation of COX-2 mRNA and protein levels. By causing the inhibitory protein IκBα to get phosphorylated and then degrade, TiO_2_ NPs induced both the nuclear translocation and DNA binding of nuclear factor-kappaB (NF-κB) in PDL cells. When treated with TiO_2_ NPs, extracellular signal-regulated kinase (ERK)1/2 and Akt was quickly activated. These proteins may sit before NF-κB. Giving PDL cells the MEK1/2 inhibitor U0126 and the PI3K inhibitor LY294002 greatly reduced the activity of NF-κB and the production of COX-2 when TiO_2_ NPs were present. When PDL cells were treated with TiO_2_ NPs, more ROS gathered inside the cells. Adding the ROS scavenger N-acetyl cysteine (NAC) to cells before adding TiO_2_ NPs stopped the particles from increasing the expression of p65, p50, and COX-2 [112]. To eliminate oral biofilm, magnetically activated NPs are of interest. Specifically, iron oxide NPs (IONPs) may be functionalized as antimicrobial particles and are remotely controlled by magnetic fields. In order to ascertain the safest, most effective IONPs size ranges and treatment concentrations of active magnetic NPs for the removal of dental biofilms, R. Fritz et al. present data in multi-species bacterial cultures, established biofilms, human gingival keratinocytes, and human gingival fibroblast cells alone and in the presence of multispecies biofilm co-cultures. Researchers find that IONPs coated with alginate (ATA) are more effective than those coated with dextran and that smaller diameters (~ 8 nm as opposed to > 20 nm) seem to have better antibacterial activity. In an in-vitro periodontitis model, human gingival keratinocyte (TIGK) cells co-cultured with treated and untreated multispecies biofilms likewise showed a tendency of decreased inflammatory markers in wells with IONP-treated biofilms [113].

**Table 1:** Metal and metal oxide nanoparticles antibacterial mechanism and common production method

Metal NPs	Antibacterial mechanism	Common production method	Refs
AuNPs	By causing the formation of perforations in the bacterial cell wall, AuNPs exert their antibacterial effect, manifesting in cell demise and the subsequent loss of cellular contents	One of the most well-known methods for the synthesis of AuNPs, the Turkevich method, was developed by Turkevich in 1951 and relies on the reduction of HAuCl4 by citrate in water	[114, 115]
AgNPs	AgNPs are capable of penetrating bacterial cell walls, thereby altering the configuration of cell membranes and potentially inducing cellular demise. By releasing silver ions, they are capable of increasing the permeability of cell membranes, generating reactive oxygen species (ROS), and interfering with the replication of deoxyribonucleic acid	The categorization of current synthesis methods can be delineated into two distinct types: bottom-up and top-down. The top-down approach involves the utilization of diverse physical forces—including mechanical influences (e.g., crushing, grinding, and milling); electrical forces (e.g., electrical arc discharge or laser ablation); and thermal forces (e.g., vapor condensation—to generate metal NPs from bulk materials. The bottom-up approach involves the nucleation and proliferation of molecular components to form complex aggregates. Chemical and biological synthesis are prevalent bottom-up approaches to produce NPs from precursor salts	[116, 117]
MNPs	The antibacterial mechanisms exhibited by magnetic nanoparticles (MNPs) are believed to result from two factors: Activation of ROS on the surfaces of the NPs induces oxidative stress within the bacterial cell, leading to cellular demise	The process of producing Fe2O3-NPs was carried out via hydrothermal means. The procedure entailed the dissolution of 0.85 mg of FeCl3.6H2O in 100 mL of double-distilled water within a 250 mL round-bottom flask. This was followed by 45 min of magnetic agitation at 85 °C at 800 rpm	[118, 119]
CuNPs	CuNPs are exceptionally reactive by their high surface area to volume ratio, which enables them to interact profusely with the cell membrane, thereby causing cell mortality by damaging cellular genetic materials	The present inquiry pertains to the environmentally friendly production of CuNPs using two distinct techniques: (I) a time-based approach and (II) thermal treatment of an aqueous solution. The plant extract in question is Moringa oleifera Lam	[120, 121]
TiONPs	The antimicrobial action of TiO_2_ is frequently attributed to ROS that generate a charge-inducing charge in the presence of O2 due to band-gap irradiation. ROS kills bacterial cells via a variety of mechanisms by which they react	Molten matrix sputtering (MMS) produced a transparent resin comprising titanium oxide NPs. To acquire homogenous dispersions of NPs, the low vapor pressure of the liquid pentaerythritol ethoxylate (PEEL) substrate enables the direct application of this vacuum technique to liquid PEEL while agitating	[122, 123]
ZnO NPs	The antibacterial properties of ZnO NPs are appealing, which can be attributed to their increased specific surface area and improved particle surface reactivity resulting from the reduced particle size	ZnO NPs were synthesized by a hydrothermal process employing sodium hydroxide and Zn nitrate hex-hydrate	[124, 125]

## Green synthesis of metals and their oxide nanoparticles in periodontitis

Compared to conventional approaches, the mass-produced by GS of NPs using live cells through biological processes is greater. Many biochemicals and components used to stabilize and reduce green NPs originate in plants [126]. The NPs' GS may be broken down into three distinct classes: extracellular, intracellular, and phytochemical. Metal NPs may be synthesized using various plant components; this process is inexpensive, safe for the environment, and efficient. Compared to physical and chemical alternatives, green-synthesized NPs are more effective in removing colors, antibiotics, and metal ions [127]. When it comes to creating NP particles, the GS approach is superior since it helps lower toxicity, improve stability, is environmentally benign, and saves money. It is widely accepted that the secondary metabolites (polyphenols) produced by plants play a crucial role in developing the GS of NPs. The GS method is more refined, secure, economical, replicable, stable, and adaptable than competing methods [48, 128]. *Staphylococcus* is the most prevalent bacterium responsible for microbial infections associated with biofilms. However, other bacteria, including *Streptococcus mutans*, *Porphyromonas gingivalis*, and *Aggregatibacter actinomycetemcomitans* have also been linked to the development of periodontitis [129]. The fundamental goal of periodontal therapy is to eliminate the bacterial biofilm and reduce inflammation so that the illness may be controlled effectively. In the past, several different antimicrobial drugs were used to treat periodontal disease. Therefore, it is essential to look into a wide range of agents with cutting-edge physicochemical properties, with a particular focus on antibacterial agents that have novel and distinctive qualities that might be used as a replacement for periodontal therapies [130, 131]. NMs as therapeutics have recently emerged as an innovative approach to halting the progression of many devastating illnesses. Extreme reaction monitoring has been used by researchers to differentiate between biochemical reduction of NPs and standard manufacturing methods. The cytotoxicity of green NPs was much lower than that of chemical NPs, indicating that they are safe for usage in a variety of biomedical settings [132] (Table 2) (Fig. 5).

**Table 2:** Therapeutic efficiency of green synthesis metals and their oxide nanoparticles in periodontitis

Metal and metal oxide nanoparticles	Effects	Green synthesis method	Refs
TiO_2_ NPs	The antibacterial and antibiofilm qualities of G-TiO_2_ NPs were studied about *S. mutans*, *Citrobacter freundii*, and *Candida albicans*. This work shows that TiO_2_ NPs manufactured sustainably have exceptional antibacterial and antibiofilm properties	TiO_2_ NPs were green-synthesized using extracts from *Azadirachta indica twigs*, *Ficus benghalensis*, *Syzygium aromaticum*, *Mentha arvensis*, *Citrus aurantifolia*, *Echinacea purpurea*, and *Acanthophyllum laxiusculum*	[133]
CuNPs	CuNPs are a promising option for usage as an anti-peri-implantation agent in dental implants due to their bactericidal effect against *Aggregatibacter actinomycetemcomitans* (one of the primary pathogens responsible for generating localized aggressive periodontitis) and their cytocompatibility	One of the most common approaches for producing Cu and CuO NPs involves combining a known concentration of the plant extract with an available precursor concentration, heating the combination to a specified temperature, and continuously stirring the mixture at a predetermined duration. For example, these extracts have come from plants including *Celastrus paniculatus*, *Cardiospermum halicacabum*, and *Zingiber officinale*	[134–136]
IONPs	The results showed that the synthesized BEP-IONPs exhibit potent antibacterial action, with a high MB dye adsorption capacity of up to 92.7% at 210 min and a zone of inhibition of 23.5 mm for gram-negative bacteria *P. aeruginosa*	Researchers in their study, Brown Egyptian Propolis (BEP) extract was used in the synthesis of IONPs because of its reducing and stabilizing properties	[137]
AgNPs	The current work finds that biogenic AgNPs manufactured using EFLAE have a high potential for inhibition against microbiota pathogens that generate periodontitis, including *E. Coli*, *B. cereus*, *S. pyogenes*, and *P. aeruginosa*	Investigators in the study made biosynthetic AgNPs using an aqueous extract from Erythrina fusca leaves (EFLAE)	[138]
AuNPs	BCL-AuNPs at a sub-MIC concentration demonstrated noteworthy anti-biofilm efficacy against *P. aeruginosa* PAO1. A decrease in biofilm formation of 58.74 ± 5.8% and 76.51 ± 4.27% was observed using the microtiter plate assay and tube method, respectively, in response to treatment with BCL-AuNPs at a concentration of 100 g mL − 1	The spherical AuNPs were synthesized using the phytocompound baicalein as a capping and reducing agent, as demonstrated by the researchers	[139]
ZnO NPs	The antibacterial activity of ZnO NPs-containing composite resin on *S. mutans* was much greater than that of AgNPs-containing composite resin	ZnO NPs may be synthesized from phenols and flavones, two plant extracts	[136, 140–142]
Bi_2_O_3_NPs	Oral antiseptics have been shown to exhibit comparable effects to these NPs in the conducted experiments. The introduction of zerovalent BiNPs halted *S. mutans* biofilm production entirely	Bi_2_O_3_ NPs derived from plant extracts are extracted from various tree parts, including the bark, roots, leaves, flowers, fruit extracts, and shells	[143, 144]

### AuNPs

Many different physical and chemical techniques have been explored and/or used in producing AuNPs. Many recent articles have focused on the synthesis and characterization of AuNPs because of their distinctive physicochemical properties and extensive range of applications. AuNPs produced through physical means (such as laser ablation) have a narrow particle size distribution, but the yield is poor, and the cost is high. Chemical methods (such as using sodium borohydride) exist for producing AuNPs. Alternative green methods were developed because of the harmful effects of organic solvents and reducing reagents used in the chemical manufacturing of AuNPs. NMs known as AuNPs may be easily manufactured by a single-step green chemical procedure. They are well-known for being non-toxic and biocompatible. Due to their properties, AuNPs are a promising candidate for usage in the biological sciences [136, 145, 146]. Plants known as "hyperaccumulators" can scavenge metals from the environment. A portion of the biomass in an alfalfa plant's leaves and stems may contain atoms of Au. For efficient and rapid extracellular production of Au, Ag, and CuNPs, many plant broth extracts have been used and reported on in recent years. These include neem, *Aloe vera*, *Arena sativa*, alfalfa, wheat, geranium, *Hibiscus sabdariffa*, and lemongrass. It has distinct nanoscale Au characteristics, and its many functions make it well-suited for therapeutic use and widespread use in nanobiotechnology. Due to their nanostructures, enormous surface volume, and biocompatibility, AuNPs have been employed to treat gum problems, dental cavities, tissue engineering, and dental implantology. Due to their antimicrobial and antifungal properties, AuNPs are used to increase the efficacy of various biomaterials. They come in various sizes and concentrations to demonstrate their medicinal benefits. Due to their desirable characteristics, AuNPs have the potential to be used as fillers in biomaterials. Diagnosis of periodontal disease is essential for halting its progression and beginning effective therapy. Due to their important optical features, AuNPs play a crucial role in detecting periodontal disease. The results suggest that the size and concentration of AuNPs influence the growth of these cells in a beneficial way. Therefore, tissue engineers may employ AuNPs as a resource to aid in the repair of damaged or sick tissues [136, 147, 148]. When NPs are being made by living things, all of these biological chemicals can lower the amount of Au^3+^ present. Most plant parts, like leaves, flowers, roots, and seeds, can help plants grow again [149]. Terpenoids, vitamins, polysaccharides, proteins, amino acids, alkaloids, (poly) phenolic compounds, aromatic amines, tannins, saponins, ketones, aldehydes, flavonoids, organic acids, and enzymes are just a few of the many biomolecules and metabolites found in leaf extracts. These substances function as RAs and stabilizers of nanosuspensions during the phytosynthesis process. The primary phenolic chemicals found in plants, namely flavonoids like proanthocyanidin, kaempferol, quercetin, and genistein, are thought to be in charge of producing AuNPs [150]. Environmentally friendly NP production utilizing biological molecules extracted from plants outperformed conventional chemical processes. The assembly processes of these plant-based NPs are highly controlled, which makes them excellent candidates for the fabrication of metal NPs [151]. Since two thousand years ago, *Morinda citrifolia* has been recognized for its medicinal properties. Primarily cultivated for its roots, foliage, and fruits, this plant, which originates in Tropical Asia, appears to be a highly esteemed medicinal specimen with extensive folk medicine use. The current study documents how AuNPs were synthesized utilizing an aqueous extract of *Morinda citrifolia* roots. In the UV–vis spectrum, the synthesized AuNPs were distinguished by a peak at 540 nm. Protein-containing extract may have been responsible for the formation of the NPs and may have played a significant role in the stabilization of the formed NPs, according to the FTIR result [152]. For the first time, Scrophularia striata (SS) extract was used in this study's quick, affordable, and environmentally friendly method to create SS-AuNPs by reducing, capping, and stabilizing the material. Against the examined microorganisms, the prepared SS-AuNPs demonstrated strong antibacterial activity. The MIC for the strains of *S. aureus*, *Enterococcus faecalis*, *P. aeruginosa*, *Acinetobacter baumannii*, *E. coli*, *K. pneumonia*, and *Proteus mirabilis* were found to be 11.875, 95, 47.5, 47.5, 23.75, 11.875, and 47.5 µg/ml [153]. To evaluate the success of implant therapy, bone regeneration (osteointegration) is a fundamental principle. The objective of wong et al. investigation was to evaluate the analgesic and osteoinductive properties of AuNPs synthesized with phytochemicals derived from *Anogeissus latifolia* (*A. latifolia*). The AuNPs synthesized by Green exhibited remarkable stability in various blood components, including bovine serum albumin (2%), human serum albumin (2%), cysteine (0.2 M), and histidine (0.2 M). When the biofabricated AuNPs were examined using erythrocytes and periodontal fibroblasts, respectively, for blood compatibility and cytocompatibility, it was also shown that the substances were not dangerous. When exposed to MG-63 cell lines, AuNPs exhibited a higher percentage of cell viability (138 ± 27.4) than the control group (96 ± 3.7), indicating their potential for osteoinduction. Additionally, analgesic activity experiments showed that the generated AuNPs and the aqueous leaf extract of *A. latifolia* exhibited a substantial antinociperceptive effect. Researchers showed that the stable, biocompatible, and environmentally friendly AuNPs were used as a bone-inducing agent during dental tissue implantation treatments and as an effective analgesic property for pain management in nursing care [154].

As a result of its anti-inflammatory, antipyretic, and anti-hyperglycemic characteristics, *Pterocarpus santa*, also known as red sandal, *yerra chandanam*, or *lal chandan*, is one of the world's most costly plants. The purpose of this research was to create a synthetic version of red sandal AuNPs and to define and evaluate their antioxidant and anti-inflammatory activities; color change and UV–visible spectroscopy were used to track AuNPs' formation, and transmission electron microscopy (TEM) examination confirmed that the Pterocarpus santa-mediated AuNPs are of spherical shape and measure between 2 and 35 nm in size. At a concentration of 50 g/ml, biosynthesized red sandal AuNP inhibited DPPH radical activity by 83%. Inhibition and protection were highest (80.5%) at 50 g/mL for red sandal AuNP. Good antioxidant and anti-inflammatory effects were observed in AuNPs synthesized through red sandalwood GS, suggesting their potential for use in regenerative periodontal treatment [155]. An environmentally friendly microwave-assisted production of colloidal Ag° and AuNPs utilizing plant extract from Oroxylum indicum (Oi) is highlighted in the research. The flavonoids found in plant extracts serve as reducing and stabilizing agents during the production of Oi-AuNPs and Oi-AgNPs. Through the use of mass spectrometry and NMR, the presence of flavonoids in plant extracts of Oi was verified. According to HRTEM examination, the particles' sizes were 5.25 nm ± 1.00 nm (AuNPs) and 15 nm ± 3 nm (AgNPs). Oi-Ag and Oi-AuNPs that are produced as a consequence have FCC. The average particle size was determined using Debye–Scherrer’s equation as 21 nm and 8 nm, respectively. It is discovered that the produced Oi-Ag and Oi-AuNPs are very poisonous to the bacterium *S. aureus* and *E. coli*. Additionally, the reduction of 4-nitrophenol was used to examine the catalytic efficiency of the produced Oi-Ag and Oi-AuNPs. According to the obtained kobs values, which were 0.12 s-1 for AgNPs and 0.22 s-1 for AuNPs, AgNPs are twofold less active than AuNPs. The findings show that the microwave-assisted GS of Ag and AuNPs created is safe and effective against bacteria. It may be used for two purposes, one of which is reducing 4-NP. For the 4-NP reduction study, the reaction kinetics and catalyst recycling efficiency were also analyzed. Finally, the Ag and AuNPs produced by microwave-assisted biosynthesis show great promise as catalysts for hydrogenation processes and highly effective antibacterial agents [156]. Utilizing AuNPs via the GS is an ecologically sustainable approach that holds promise for microbistatic and microbicidal applications. The present investigation centers on the synthesis of AuNPs at room temperature via the aqueous leaf extract of Justicia glauca and the treatment of chloroaurate ions, which demonstrate an antagonistic effect against oral pathogenic bacteria and fungi (*Micrococcus luteus* (*M. luteus*)*, B. subtilis*, *S. aureus*, *S. mutans*,* Lactobac*). The ions are treated with azithromycin (AZM) and clarithromycin (CLR) antibiotics. Regarding dimension, the biosynthesized AuNPs were spherical, hexagonal, and stable to within 32.5 ± 0.25 nm. Against oral pathogens, the AuNPs and drug-conjugated AuNPs exhibited prospective antibacterial and antifungal activity. Biogenic AuNPs showed MIC values ranging from 6.25 to 25 μg/mL when tested against a specific set of oral pathogens. In summary, we deduce that the biogenic drug delivery system utilizing AZM and CLR holds promise as a prospective antimicrobial treatment, contingent upon a comprehensive evaluation of its cytotoxicity in vitro and in vivo [157]. The spherical AuNPs were synthesized using the phytocompound baicalein as both a capping and RA, as demonstrated by the researchers. They were characterizing and assessing the anti-biofilm effectiveness of baicalein-coated AuNPs (BCL-AuNPs) against *P. aeruginosa* PAO1. The average dimension of the biosynthesized BCL-AuNPs was found to be 26.5 nm, and they exhibited a spherical morphology. BCL-AuNPs at a sub-MIC concentration demonstrated noteworthy anti-biofilm efficacy against *P. aeruginosa* PAO1. A decrease in biofilm formation of 58.74 ± 5.8% and 76.51 ± 4.27% was observed using the microtiter plate assay and tube method, respectively, in response to treatment with BCL-AuNPs at a concentration of 100 g mL − 1. An observed decrease in exopolysaccharide (EPS) production of 81.29 ± 2.96 percent was significant. Additionally, the swimming and swarming behaviors were significantly impeded when BCL-AuNPs were present. The impact of BCL-AuNPs on the surface topography and architecture of *P. aeruginosa* biofilm was further investigated using light microscopy and CLSM. Therefore, the results of the investigation raise the possibility of using BCL-AuNPs in the creation of new treatments for the avoidance and treatment of chronic illnesses caused by biofilms [139].

### AgNPs

AgNPs are a novel and developing field against dangerous bacteria. AgNPs are widely investigated in the structural range of 1–100 nm. Additionally, nano-Ag has a significant accessible surface area that permits the binding of any ligands. AgNO_3_ is typically employed in the form of antimicrobial activity [158]. Numerous mechanisms of action have been proposed to explain the antibacterial activities of AgNPs. These include the capacity of AgNPs to adhere to bacterial cell walls and induce structural modifications in the cell membrane, the ability to disrupt and permeabilize the cell membrane through the generation of reactive AgNP radicals, and the release of Ag ions within the intracellular environment, leading to the impairment of various cellular functions. In the past, the synthesis of AgNPs mainlyincluded a chemical process whereby AgNO_3_ was reduced using a chemical RA. Various environmental resources, including bacteria, plants, algae, and fungi, use organic processes. The synthesis of AgNPs using microbes has a high potential for scalability and offers inherent environmental benefits. However, it is worth noting that the production process using microorganisms is comparatively more costly than plant extracts [159]. Plant extracts are used to synthesize AgNPs because of their rich content of flavonoids, polysaccharides, sapogenins, and steroids. These compounds serve as RA and capping agents, effectively preventing the aggregation of NPs and enabling enhanced control over their size. Typically, the process of acquiring AgNPs from plant extracts is considered to be a relatively simple technique. The collection of plant fragments is followed by the purification of sterile water, further drying in a shaded environment, and, ultimately, pulverization. The desiccated powder is boiled in deionized water to produce the botanical extract. The resultant infusion undergoes filtration to eliminate any insoluble constituents. The solution, which has a concentration of 1 mM AgNO_3_, is then augmented with a specific volume of the plant extract. The change in color of the medium, often resulting in a dark brown hue, together with the observation of ultraviolet–visible (UV–Vis) spectra, may serve as a means to validate the synthesis process of AgNPs. AgNPs may be efficiently collected by performing several centrifugation processes at a speed of 12,000 rpm for 15 min [160].

The goal of this study is to conduct GS of AgNPs and to test them against the harmful microbes that bring on periodontal disease. Reasons for this include proof that bacteria play a significant role in periodontal disease, *Erythrina fusca's* ability to alter the human pathogen microbiota, and the ability of eco-friendly AgNPs to enhance the body's natural antimicrobial response. Investigators in the study made biosynthetic AgNPs using an aqueous extract from Erythrina fusca leaves (EFLAE). They were then optimized, characterized, and tested for stability. Their antimicrobial potential against pathogenic human microflora that cause periodontitis (*P. aeruginosa*, *E. coli*, *S. pyogenes*, and *S. aureus*) was assessed. The brown coloring and surface plasmon resonance signal at 433 nm were the main factors contributing to AgNP GS's success. The parameters for AgNP biosynthesis were 5 mM AgNO_3_ concentration, 1:9 EFLAE and AgNO_3_ volumetric ratio, pH 7, 60 °C temperature, and 2 h, using UV–visible spectrometry-driven optimization. According to stability studies, a signal that supports AgNP stability appears between 415 and 424 nm. Changes and expansions in character studies were acknowledged X-ray diffraction (XRD) signals at 38.95, 44.97, 64.92, and 78.97 representing 111, 200, 220, and 311 AgNPs cubic structure planes; elemental Ag 83.66%, carbon 11.87%, and oxygen 4.47% in energy-dispersive X-ray spectroscopy; and AgNPs size below 32 nm in field emission scanning electron microscopy micrograph. When tested using the suitable diffusion method against pathogenic microflora that cause periodontal disease, optimized and characterized biogenic AgNPs showed the largest inhibitory zone (measured in millimeters) against *B. cereus* (13 and 18), followed by *P. aeruginosa* (11 and 19), *E. coli* (10 and 18), and *S. pyogenes* (9 and 15) at administered doses of 50 and 100 µg/ml. The current work finds that biogenic AgNPs manufactured using EFLAE have a high potential for inhibition against microbiota pathogens that generate periodontitis, including *E. Coli*, *B. cereus*, *S. pyogenes*, and *P. aeruginosa*. It also suggests EFLAE as a possible source for AgNPs GS [138]. Another study demonstrated that *Streptomyces rochei* MS-37 may be the first of its kind as a novel marine actinobacterium capable of green biosynthesis of AgNPs with promising antimicrobial, anti-inflammatory, anti-biofilm, and antioxidant candidate properties for preventing dental membrane-associated infections. In this investigation, we employed *Streptomyces rochei* MS-37, a novel marine actinobacterial strain, to biosynthesize Ag NPs for many potential medical uses. The AgNPs had a peak at 429 nm in surface plasmon resonance spectroscopy. The AgNPs were spherical, very stable (26 mV), extremely small (average 23.2 nm by TEM, 59.4 nm by DLS), and protected by capping agents. The MICs of the AgNPs exhibiting antibacterial activity varied from 8 to 128 µg/mL. Bacterial adherence and bacterial penetration across guided tissue regeneration membranes were qualitatively evaluated using periodontal pathogens. The results indicated that AgNPs could help prevent membrane-associated infection. AgNPs were also very effective in scavenging nitric oxide free radicals. They had a significant anti-denaturation impact when their anti-inflammatory function was evaluated by inhibiting protein denaturation. Compared to human peripheral blood mononuclear cells (PBMCs), CAL27 was shown to be more sensitive to the cytotoxicity of the AgNPs, with IC50 values of 34.03 µg/mL for the former and 81.16 µg/mL for the latter. Anti-inflammatory properties of AgNPs have been shown by their ability to inhibit protein denaturation and scavenge free radicals with the help of nitric oxide. The results of this work pave the way for the use of marine actinobacteria in the biosynthesis of Ag NPs, which has promising biological applications, including periodontal therapy [132, 161, 162].

Different research discovered that using the leaf extract of Justicia glauca enhanced the GS of AgNPs. The water-soluble organics in the leaf extract are primarily responsible for converting AgNO_3_ solution to AgNPs. The sizes of the AgNPs were between 10 and 20 nm, according to TEM scans. Tested for their antimicrobial properties against green synthesized AgNPs and drug-blended AgNPs were *S. mutans*, *S. aureus*, *Lactobacillus acidophilus* (*L. acidophilus*), *M. luteus*, *B. subtilis*, *E. coli*, *P. aeruginosa*, and *C. albicans*. These bacteria have all been related to dental caries and periodontal disease. The antibacterial and antifungal activity of the AgNPs and drug-blended AgNPs is striking. Against some bacteria linked to dental cavities and periodontal disease, AgNPs were shown to have a MIC of 25–75 μg/mL. This research indicates that J. glauca leaf extract may perform GS of AgNPs outside of cells. Antimicrobial activities of green synthesized AgNPs and drug-blended AgNPs against *S. mutans*, *S. aureus*,* L. acidophilus*,* M. luteus, B. subtilis*,* E. coli*,* P. aeruginosa*, and *C. albicans*, the causative agents of dental caries and periodontal disease, have been assessed [163].

The purpose of this research was to describe certain chemicals present in lyophilized hydroalcoholic extracts of *S. cumini* seed and flower as well as AgNPs-HEScSeed and AgNPs-HEScFlower and evaluate their antibacterial activities. Seven and seventeen phytochemical substances were found in HEScSeed and HEScFlower, respectively. Stable, plant-extract-AgNPs were observed to have varying sizes and forms. Extracts and AgNPs-plant extracts showed antibacterial activity (648.45,187.5 μg/mL and 31.22,000 μg/mL, respectively) against all species tested (*A. naeslundii*, *C. albicans*, *F. nucleatum*, *S. aureus*, *S. epidermidis*, *S. mutans*, *S. oralis*, and *V. dispar*). Medically and dentally relevant infections show species-specific MICs and minimum inhibitory concentrations (MMCs) for the extracts of *S. cumini* seed and flower. The bacteriostatic and fungistatic effects of AgNPs-HEScSeed and AgNPs-HEScFlower may be achieved at much lower concentrations than those of plant extracts due to their unique forms, sizes, chemical components, stability, and electronegativity (capping). The growth of *S. aureus* (diameter of the zone of inhibition: 1.40 ± 0.07 mm), *E. coli* (2.75 ± 0.35 mm), *C. albicans* (2.25 ± 0.07 mm), and *S. Typhimurium* (1.45 ± 0.14 mm) was inhibited when these AgNPs were screened for antimicrobial action (disc diffusion method) against some human pathogenic strains. [40].

The medication delivery product made by GS of NPs utilizing Ocimum Sanctum is quick, cheap, and environmentally friendly. Pure Ocimum sanctum extract (OSE), AgNO_3_, and AgNPs synthesized with OSE (AgNP) were found to have antimicrobial activity against four periodontal pathogens (*Fusobacterium nucleatum*, *Porphyromonas gingivalis*, *Aggregatibacter actinomycetemcomitans*, and *Prevotella intermedia*) using MIC and minimum bactericidal concentration (MBC) tests. All test solutions seem to be dose-dependent in their sensitivity to the test microorganism, as seen by the findings. The antibacterial effect may, therefore, be enhanced by increasing the concentration. Based on these results, it seems that AgNP has more potent antibacterial action than previously thought and that *A. actinomycetemcomitens* is more vulnerable to this agent. A new paradigm for treating chronic periodontitis may emerge from the development and clinical use of AgNPs synthesized with *Ocimum sanctum* as a regulated local drug delivery system used in conjunction with current best practices. This has the potential to help us beat the adverse outcomes of using synthetic drugs and the problem of drug resistance that exists today [164].

*S. rochei* MS-37 may be the first marine actinobacterium to have its performance studied about the GS of AgNPs and its potential as antibacterial, anti-inflammatory, anti-biofilm, and antioxidant options for decreasing membrane-associated dental illnesses. In this research, the novel marine actinobacterial strain *S. rochei* MS-37 was employed to biosynthesize AgNPs with potential medicinal uses. The AgNPs had a peak at 429 nm in surface plasmon resonance spectroscopy. The AgNPs were spherical, very stable (26 mV), extremely small (average 23.2 nm by TEM, 59.4 nm by Dynamic Light Scattering (DLS)), and protected by capping agents. AgNPs with promising antibacterial activity have MICs between 8 and 128 g/mL. Qualitative assessments of microbial adherence and bacterial penetration across guided tissue regeneration membranes were performed using periodontal pathogens. The results indicated that AgNPs could help prevent membrane-associated infection. Additionally, it was shown that the AgNPs exhibited a substantial anti-denaturation effect and a high capacity for scavenging nitric oxide free radicals when their anti-inflammatory function was evaluated using these methods. The IC50 values for the AgNPs were 81.16 µg/mL in human PBMCs and 34.03 µg/mL in CAL27, respectively. The results of this research pave the way for the use of marine actinobacteria in the biosynthesis of AgNPs, which has promising biological applications, including periodontal therapy [132].

### IONPs

Iron NPs (INPs) are fascinating because of their unique physicochemical properties, including their strong magnetism, microwave absorption capabilities, low toxicity, and high catalytic activity. There are three main types of iron NPs (INPs): iron oxide (Fe_3_O_4_) NPs, iron oxide-hydroxide (FeOOH) NPs, and zero-valent iron (ZVI) NPs [165]. The ecologically friendly and cost-effective therapeutic approach of INP green production is gaining favor. Because of their low environmental impact and high cost-effectiveness, green processes have replaced traditional methods for synthesizing INP from *Citrus sinensis* plant-mediated extract. The antibacterial effects of biologically produced IONPs were studied using Gram-negative and Gram-positive bacteria. These results showed that IONPs had considerable antibacterial potential by inhibiting many bacterial species [42]. IONPs such as magnetite and maghemite have attracted a lot of attention lately. Gao et al. reported a unique approach to managing biofilms (plaques): catalytic (CAT) NPs constructed of biocompatible Fe_3_O_4_ with the peroxidase-like activity that promotes extracellular matrix breakdown and bacterial mortality within acidic niches of caries-causing biofilms. It has been shown that the synergy of INP enhances the antibacterial action of both natural and synthetic drugs. It investigated how common natural materials, such as clove buds, neem leaves, and green tea leaves, affected *S. mutans.* Researchers employed three different plant extracts: one with INP, one with INP and amoxicillin, and one with only the plant extract to examine the effects of different treatment combinations on *S. mutans*. The combination of INP with an antimicrobial agent increases the antimicrobial agent’s effectiveness [136]. It has been shown that the synergy of INP enhances the antibacterial action of both natural and synthetic drugs. It investigated how common natural materials, such as clove buds, neem leaves, and green tea leaves, affected *S. mutans*. Researchers employed three different plant extracts: one with INP, one with INP and amoxicillin, and one with only the plant extract to examine the effects of different treatment combinations on *S. mutans*. The combination of INP with an antimicrobial agent increases the antimicrobial agent’s effectiveness. Both the cold seed extract (S-MNP) and the pulp extract (P-MNP) MNPs were round after they were manufactured. The S-MNP had a narrower size range of 6–15 nm, whereas the P-MNP ranged from 12 to 45 nm. Both particles were just as effective as one another in killing off the bacteria investigators put them through their paces with. Bacterial growth was decreased by 16.0–99.0% and 10.0–91.0% by S-MNP and P-MNP, respectively, at concentrations of 0.48–1000 μg/Ml for Gram-positive and negative bacteria. With a maximal inhibition of 63–88% following treatment with S-MNP and P-MNP (1 mg/Ml), respectively, *C. albicans* was the least impacted microbe. CTC’s aqueous extract may be utilized to make MNPs have antibacterial properties. The presented methods are straightforward and may be used for widespread GS of MNPs [166]. Researchers, for the first time, use *A. concinna* fruit extract in producing magnetite NPs (Fe_3_O_4_ NPs), which is described as being environmentally friendly, straightforward, and effective. The extract of the fruit of *A. concinna* is used as a preservative and stabilizer. UV–visible absorption spectra (UV–Vis-NIR) are used to investigate whether or not *A. concinna* fruit extract may inhibit the reduction of Fe3 + ions. XRD supports the cubic spinel structure of Fe_3_O_4_ NPs, and the average crystallite size of the produced NPs is determined to be 28 nm. Scanning electron microscopy (SEM) images of Fe_3_O_4_ NPs, used for morphological investigations, show that the particles have a quasi-spherical shape. Biomedical uses for the green synthesized Fe_3_O_4_ NPs have been confirmed by their significant antibacterial activity against gram-negative microorganisms such as *E. coli* and *P. aeruginosa* [167]. Metal oxide NPs have found several applications, such as antibacterial agents, anticancer medications, wastewater treatment agents, and degradants for hazardous organic dyes. Researchers in their study, Brown Egyptian Propolis (BEP) extract was used in the synthesis of IONPs because of its reducing and stabilizing properties. After being manufactured, BEP-IONPs were put to use to eliminate germs and get rid of cationic methylene blue (MB) dye in water. The average particle sizes of the spherical BEP-IONPs synthesized with a 1:1 (BEP: FeCl3) ratio and those created with a 1:2 (BEP: FeCl3) ratio were around 87 and 194 nm, respectively. Several gram-positive and gram-negative bacterial strains, including *B. subtilis*, *P. aeruginosa*, *S. aureus*, and *E. coli*, were used to assess the antibacterial activity. The results showed that the synthesized BEP-IONPs exhibit potent antibacterial action, with a high MB dye adsorption capacity of up to 92.7% at 210 min and a zone of inhibition of 23.5 mm for gram-negative bacteria *P. aeruginosa*. With a constant rate of 0.0178 min − 1, the photocatalytic degradation of MB dye by BEP-IONPs was shown to follow pseudo-first-order kinetics. Ultimately, the produced BEP-IONPs have the potential to function as both photocatalysts for the elimination of hazardous organic dyes and as efficient antibacterial agents against human diseases [137]. An AgNP system based on Ulvan was tested for antibacterial efficacy in a separate investigation. Ulvan, a sulphated polysaccharide isolated from Ulva lactuca, was used to carry out the GS of biogenic AgNP. An experimental mouthwash containing AgNPs was evaluated for its safety and effectiveness. The existence of AgNPs was confirmed using spectrophotometric measurement (UV-A visible spectrophotometer), and their characterization was confirmed using Fourier transform infrared spectroscopy (FTIR), XRD, and TEM. AgNPs at 50 μL/mL inhibited 93.15% of the antioxidant activity in a DDPH assay. Against *S. mutans*, *S. aureus*, *Lactobacillus*, and *C. albicans*, the mouth rinse containing AgNPs showed antibacterial action at a concentration of 100 µL/mL. This research suggests that mouthwash made using the Ulvan-AgNP system may be an effective, safe, and antibacterial agent in the mouth [168].

### CuNPs

In the last two decades, CuNPs have received a lot of interest due to their relatively easy construction and the fact that they exhibit a wide variety of potentially useful physical characteristics that vary with their size, shape, and composition. Clean, bactericidal water is produced by Vital Water in Containers by destroying various bacterial species and strains. In addition, Cuis is a cheaper alternative to other antibacterial agents as Au and Ag. It has more antioxidant activity and a longer shelf life than comparable organic antibacterial agents. Because of their unusual crystal form and high surface area-to-volume ratio, they exhibit properties in the physical, chemical, and biological realms that are hard to find anywhere else [169]. Physical and chemical processes were employed in the CuNPs' synthesis. Despite being expensive and demanding a substantial surfactant concentration, the microemulsion method remains the most frequently used chemical strategy. Physical processes that can generate NPs include laser ablation, aerosol processes, and radiolysis. However, the expensive cost of equipment and the significant energy usage make these approaches less common. Microwave irradiation allows for the formation of CuNPs even in the absence of a stabilizing agent. CuO NPs are formed when ascorbic acid is added during the production process. Plants have been used to manufacture metallic NPs due to their accessibility, low cost, low environmental impact, and absence of toxic byproducts [170]. One of the most common approaches for producing Cu and CuO NPs involves combining a known concentration of the plant extract with an available precursor concentration, heating the combination to a specified temperature, and continuously stirring the mixture at a predetermined duration. Due to their exceptional physical properties, CuNPs are used in antibiotics. Due to their disinfecting properties and matrix stability, they cover medical equipment including heat transfer systems, antimicrobial materials, superstrong materials, sensors, and catalysts [171, 172]. Plant extracts have been used in the synthesis of CuNP. These extracts have come from plants including *Celastrus paniculatus*, *Cardiospermum halicacabum*, *Zingiber officinale*, *Eryngium caucasicum*, *Plectranthus amboinicus*, *Azadirachta indica*, *Punica granatum*, *Eclipta prostrata*, *Citrus medica Linn*., and *Madhuca longifolia*. The release of ions is not the most critical factor in the bactericidal action of CuNPs, unlike Ag. The oxidation state, size, and crystalline structure of the NPs are all essential factors. CuNPs are a promising option for usage as an anti-peri-implantation agent in dental implants due to their bactericidal effect against *Aggregatibacter actinomycetemcomitans* (one of the primary pathogens responsible for generating localized aggressive periodontitis) and their cytocompatibility. Aloe vera-derived CuO NPs have been shown to have potent anti-cariogenic effects and have found widespread usage in the dentistry field [134–136]. Hawthorn fruit ethanol and water extracts were used to make Ag and CuNPs. Particle size distribution was investigated as a function of solution pH, contact duration, temperature, metal ion precursor, and hawthorn extract type. AgNPs and CuNPs were synthesized with a monodisperse size distribution and a stable average size of 60 nm and 200 nm, respectively. Extracts were analyzed for their total phenolic content and anthocyanin content. In this study, hawthorn extract was used as a stabilizer and reductant to create a green process for preparing silver and CuNPs. There have been reports of reducing Ag and Cu ions using ethanol extract from dried hawthorn fruit and water. At relatively low concentrations (below 100 mg/l), metal NPs synthesized using green methods exhibited comparable antibacterial efficacy against the designated harmful pathogens (*A. niger*, *E. coli*, and *S. cerevisiae*). The metal NPs, synthesized from inorganic salts, all had a uniform size and shape, with an average diameter of 60 nm for AgNPs and 200 nm for CuNPs. An alternative to the more common physical and chemical synthesis techniques, the reported green NP production method shows promise [173].

**Fig. 6:**
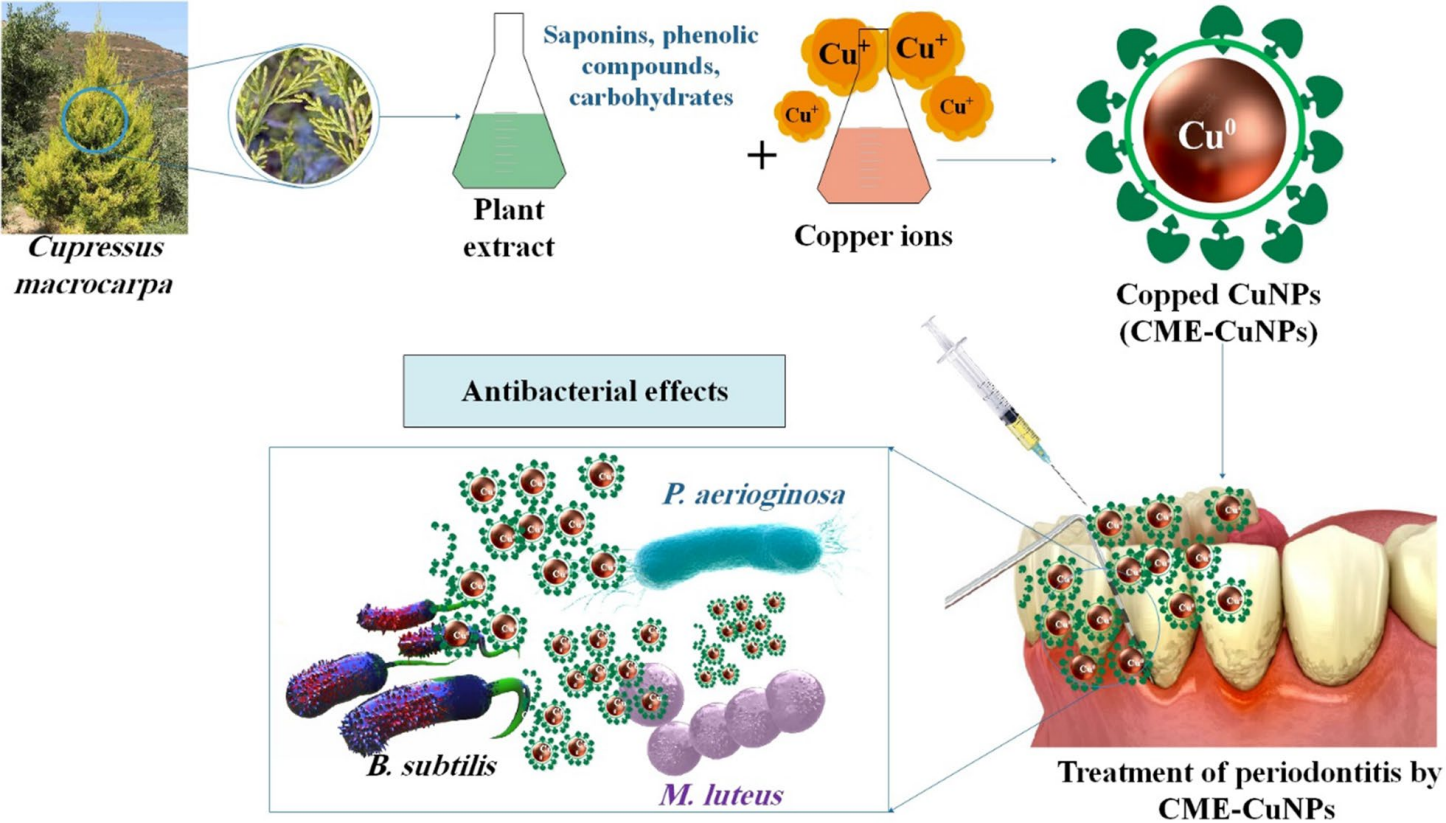
CME-CuNPs antibacterial effects on different bacteria strains in periodontitis treatment

In a different study, scientists looked at the potential antibacterial activity of CuNPs made with *Cupressus macrocarpa* extract (CME) against bacteria that cause periodontitis. Then, utilizing morphological/biochemical analysis and 16S-rRNA approaches, the antibacterial activities of CME-CuNPs were evaluated against oral microorganisms (*M. luteus*, *B. subtilis*, and *P. aerioginosa*) that cause periodontal disease. After the CME-CuNPs were described, the development of stable CME-CuNPs was indicated by the peak discovered at 577 nm using a UV–Vis spectrometer. Additionally, the effect of elliptical and spherical monodispersed CME-CuNPs with diameters ranging from 11.3 to 22.4 nm was demonstrated by the data. According to the FTIR study, the CME may include Ras, which contributed to Cu reduction and the synthesis of CME-CuNP. Additionally, the CME-CuNPs demonstrated strong antibacterial effectiveness against several isolates, surpassing the documented values in the literature. Oral bacteria were tested for their susceptibility to CME-CuNPs and the synergistic solution of clindamycin with CME-CuNPs. The solution's ability to inhibit bacterial growth was remarkable. The MIC, MBC, and fractional inhibitory concentration (FIC) of CME-CuNPs with clindamycin against the chosen periodontal disease-causing microbes were found to be between 2.6 and 3.6 μg/ml, 4–5 μg/ml, and 0.312 and 0.5, respectively. In conclusion, CME-CuNPs' synergistic antibacterial activity with clindamycin against the tested strains may be relevant for the future development of more potent medicines to manage dental illnesses. The goal of the research was to create antibacterial nano Cu using an herbal formulation of CME as an RA against microorganisms that cause infectious diseases of the periodontal region, including gram-negative *P. aeruginosa* and gram-positive *B. subtilis*. The researchers also set dental prophylaxis as their target. Investigators further assessed the antibacterial efficacy of mixing biosynthesized CuNPs with antibiotics to combat antibiotic resistance [174] (Fig. 6). *Punica granatum peel* extract was used as a capping and RA during the biological synthesis of CuNPs. Stable CuNPs were created when *P. granatum peel* extract was added to aqueous solutions of CuSO_4_·5H_2_O. The size distribution of CuNPs was determined by electron microscopy investigation to be between 15 and 20 nm. When tested in vitro against opportunistic pathogens such as *M. luteus* MTCC 1809, *P. aeruginosa* MTCC 424, *Salmonella enterica* MTCC 1253, and *Enterobacter aerogenes* MTCC 2823, the biologically produced CuNPs showed antibacterial solid activity [175].

### TiO_2_ NPs

Using Ti (IV) isopropoxide as a starting material, a straightforward precipitation process was used to create TiO_2_ NPs. Temperatures between 400 and 700 °C were used to calcine the precursor powder in the atmosphere [176, 177]. Because of their strong oxidation capabilities, high refractive index, acceptable chemical stability, reduced cost, and formidable oxidation properties, TiO_2_ NPs are well-known and adaptable oxides with increasing output [178, 179]. Furthermore, TiO_2_ NPs, along with other metal oxide NPs, have been predominantly utilized for their antimicrobial, photocatalytic, and antiparasitic properties [176, 180, 181]. One notable example is TiO_2_, which exhibits favorable biocompatibility and serves as a representative model for responding to ultrasonic irradiation to generate reactive oxygen species (ROS) for antibacterial therapy. The quantum yield of ROS in pure TiO_2_- NPs is restricted due to the large band gap and rapid recombination of electrons (e-) and holes (h +) under ultrasonic irradiation [182].

The most typical method for producing TiO_2_- NPs involves the formation of a solution of a Ti precursor with the necessary solvent. Most often, people will use ethanol or distilled water as solvents. TiO_2_ NPs (metatitanic acid or titanyl hydroxide) may be produced in an environmentally friendly manner from a variety of Ti precursors, including TTIP (Ti tetra isopropoxide), TiCl_4_ (Ti tetrachloride), and TiO(OH)_2_. One of the benefits of green nanotechnology is that TiOSO_4_ (Ti oxysulfate) and TiO_2_ bulk particles are used in the production processes. Equally applicable are water-soluble precursors [183]. The reduction and stabilization processes governing NP production are regulated by plant components such as phenolic acids, alkaloids, proteins, enzymes, and carbohydrates. Many different plant species have been used to extract TiO_2_ NPs in various forms. Rapid heating of the reaction mixture occurs when a precursor TiO_2_ salt is contaminated with the plant extract. The solution is then heated to a comfortable level and stirred continuously. A change in hue is used as a first sign of successful synthesis, and spectroscopic methods may be used to confirm this. TiO_2_ NP synthesis has been linked to various colors, from light green to very dark green [184]. The next steps include filtering, washing with distilled water, drying, and calcining the NPs. To get rid of organic groups, calcination is often done at temperatures between 400 and 800 °C. Ahmad et al. examined the antibacterial and antifungal properties of green-produced TiO_2_ NPs using Mentha arvensis leaf extract as a RA and Ti tetra isopropoxide as a precursor. Green TiO_2_ NPs have been shown to encourage antibacterial and antifungal action against various microbes. TiO_2_ NPs were green-synthesized using extracts from *Azadirachta indica twigs*, *Ficus benghalensis*, *Syzygium aromaticum*, *Mentha arvensis*, *Citrus aurantifolia*, *Echinacea purpurea*, and *Acanthophyllum laxiusculum* [136]. TiO_2_ NPs were prepared using extracts from *Ficus benghalensis*, *Syzygium aromaticum*, and twigs of *Azadirachta indica*. The antibacterial and antibiofilm qualities of G-TiO_2_ NPs were studied about *S. mutans*, *Citrobacter freundii*, and *C. albicans*. This work shows that TiO_2_ NPs manufactured sustainably have exceptional antibacterial and antibiofilm properties [133]. Plant extracts from Capsicum annum L. and Allium cepa (onion) are used in GS of TiO_2_-NPs in another work. Because of its high refractive index and its ability to absorb UV light, TiO_2_ is of interest as both a white pigment and an eco-friendly catalyst. It is inert, non-toxic, and cheap. Researchers assess NPs' antibacterial efficacy and explore the possibility of using neem and tulsi extract to boost these effects in combination treatments. Synergistic effects occur when NPs are combined with plant oil. TiO_2_ NPs containing neem and tulsi extract will soon be tested for their antibacterial efficacy against a variety of oral infections, including *S. mutans*, *L*. *acidophilus*, *S. aureus*, and *C. albicans*. TiO_2_ NPs were mixed with neem and tulsi extracts of varying concentrations to test their antimicrobial effects. The zone of inhibition for NPs against *S. mutans* was 20 mm (at conc 150 l), whereas the zone of inhibition for NPs against *C. albicans* was 18 mm (at conc 100 l). Since neem and tulsi extract-containing TiO_2_ NPs are effective against oral pathogens, including *lactobacillus* and *s. mutans*, Researchers' results confirm their efficacy as an antibacterial agent [185]. Oral bacteria and fibroblast cells were used to test the antibacterial and cytotoxic effects of green-produced new TiO_2_ NPs utilizing extracts of Iranian propolis. Propolis was collected for this investigation, and alcoholic extracts were made from the samples. Biosynthesizing the TiO_2_ NPs with propolis extracts. The FTIR study revealed that flavonoids and phenolic chemicals, in addition to TiO_2_ NPs, were present in the propolis extract. Intense bands also generated NPs, as seen by UV–Vis spectroscopy. The stabilizing ingredient was found in excellent quasi-spherical forms at roughly 21 nm, as shown by Energy Dispersive X-ray (EDX) spectra and SEM pictures. The EDX spectrum was utilized to confirm that Ti and O_2_ were present. No appreciable cytotoxic effects were seen. The data revealed that Pro1TiO_2_ (Khalkhal sample) was more effective against bacteria than Pro2TiO_2_ (Gilan sample) and TiO_2_ NPs. This research introduces a novel method for producing TiO_2_ NPs from propolis extracts, which results in a safer product and uses more accessible, environmentally friendly, and cost-effective raw ingredients. Compared to Pro2TiO_2_ NPs and TiO_2_ NPs, the performance of Pro1TiO_2_ NPs was superior. TiO_2_ NPs had the least impact. Furthermore, neither Pro1TiO_2_ nor Pro1TiO_2_ NPs inhibited *C. albicans* biofilm production appreciably. Furthermore, TiO_2_, Pro1TiO_2_, and NPs from the Gilan sample showed no significant impact on the microbial biofilm breakdown of *C. albicans*. The biofilm-reduction effects of TiO_2_ NPs were not statistically significant; however, both Pro1TiO_2_ and Pro2TiO_2_ NPs were effective against the microorganisms mentioned above [186]. In this work, investigators investigated the antibacterial activity of TiO_2_ NPs produced using Luffa acutangula leaf extract. EDX was used to determine the elemental makeup of the produced TiO_2_ NPs. *B. subtilis*, *E. coli*, *E. faecalis*, *K. pneumonia*, *S. aureus*, and *P. aeruginosa* were shown to be particularly vulnerable to the toxicity of the TiO_2_ NPs' antibacterial capabilities. The MIC was determined using a micro broth dilution experiment, and the zone of inhibition was calculated using a disc diffusion assay. Therefore, it is reasonable to assume that TiO_2_ NPs, as a unique class of antimicrobial materials, may be produced to treat microbial diseases [187].

### ZnO NPs

Zn is a widely distributed trace mineral that aids in the catalytic activity of several enzymes. Because of its biocompatibility, ZnO is used in various dental goods, including ZnO eugenol, amalgam, ceramics, and dental cement. One of the safest materials used in the pharmaceutical sector, according to the Food and Drug Administration (FDA) [188, 189]. ZnO NPs may be synthesized from phenols and flavones, two plant extracts. Biosynthesis with the help of plant extracts is a three-step procedure that is quite simple to carry out. The first step is often extracting the plant material. Then, Zn salts are introduced as a precursor to the plant extracts. At this point, metal ions are transformed into NPs before being stabilized by further additions of reducing and capping chemicals. ZnO NPs are made in the last stage of a multi-step synthesis process that includes high-temperature annealing. Studies on the development of NPs of spherical, quasi-spherical, hexagonal, rod-shaped, and agglomerate sizes and forms in *Lamiaceae* plants, including *Anisochilus carnosus*, *Plectranthus amboinicus*, and *Vitex negundo*, have been conducted at length. It was discovered that increasing the amount of plant extract decreased the average NP size. Light-induced electrostatic forces might also be responsible for the NPs' interactions with bacteria [140–142]. The antibacterial activity of ZnO NPs-containing composite resin on *S. mutans* was much greater than that of AgNPs-containing composite resin. In addition, there are several methods for quickly and easily producing ZnO NPs. The bioactivity of ZnO NPs, and hence their potential biological applications, may be enhanced, for example, by using green production methods. ZnO NP-modified implants are effective against microorganisms. Titania nanotubes and nanoleaves were coated with ZnO NPs by Elizabeth et al. Compared to unaltered nano-patterned materials, the antibacterial efficacy of the changed samples was significantly boosted [136]. *P. aeruginosa* was isolated from silt in the mangrove rhizosphere, and its biomass filtrate was utilized to biosynthesize ZnO-NPs in research. In this research, spherical ZnO-NPs with a crystalline structure and a maximum SPR (surface plasmon resonance) of 380 nm were effectively generated. The examination of the data revealed that the concentration affected these bioactivities. The ZnO-NPs, which were synthesized in a green manner, demonstrated significant effectiveness against pathogenic Gram-positive bacteria (*S. aureus* and *B. subtilis*), Gram-negative bacteria (*E. coli* and *P. aeruginosa*), and unicellular fungi (*C. albicans*). At a concentration of 200 ppm, the inhibition zones were measured at (22.3 ± 0.3 mm), (19.3 ± 0.3 and 11.7 ± 0.3 mm), and (22.3 ± 0.3 mm), respectively. The MIC values for *S. aureus* and *P. aeruginosa E. coli*, *B. subtilis*, and *C. albicans* were determined to be 200 ppm and 50 ppm, respectively, with zones of inhibition ranging from 11.7 ± 0.3 to 14.6 ± 0.6 mm. Furthermore, after 24 h, the biosynthesized ZnO-NPs exhibited a significantly higher mortality rate for Culex pipiens, with percentages of 100 ± 0.0% at 200 ppm, in comparison to Zn acetate (44.3 ± 3.3% at the same concentration and time) [190]. Investigators examined the antibacterial efficacy of ZnNPs green, which were synthesized using the microwave method in combination with Lavandula vera DC, against *Streptococcus mutans* and Actinomyces viscosus, compared to chlorhexidine. The maximal absorption wavelength of ZnNPs was between 230 and 330 nm. ZnNPs ranged in size from 30 to 80 nm, whereas most NPs were 50 to 60 nm. The optimal MICs for S. mutans and A. viscosus about ZnNPs + chlorhexidine were reported to be 1.66 and 1.66 μg/ml, respectively. Additionally, the MICs associated exclusively with ZnNPs were determined to be 13.33 and 16.33 μg/ml for *S. mutans* and *A. viscosus*, respectively. The combination of ZnNPs and chlorhexidine exhibited a statistically significant antibacterial effect (P < 0.05) against both microorganisms compared to chlorhexidine alone. The antibacterial efficacy of ZnNPs in combination with chlorhexidine was significantly more promising than that of chlorhexidine alone. However, additional research is required to elucidate the precise mechanisms and toxicity of ZnNPs [191].

### Other NPs

Au, Ag, Zn, and Ti NPs have all been the subject of extensive research due to their alleged antibacterial properties. In contrast, Bi is classified as a "green" element due to its lack of carcinogenicity and minimal propensity for bioaccumulation and cytotoxicity [192–196]. The physical mentioned above and chemical procedures necessitate using hazardous compounds and precise instruments; therefore, GS is preferable to other methods. Bio-assisted methods, known as biosynthesis or GS, provide an environmentally sustainable, cost-effective, and functional approach to manufacturing metal NPs. These methods employ biological entities such as bacteria, fungi, viruses, yeast, actinomycetes, plant extracts, and more. Biosynthesized Bi_2_O_3_ NPs are less expensive, more environmentally benign, more straightforward to manufacture, and non-toxic than those produced by microorganisms. Moreover, in contrast to Bi_2_O_3_ NPs derived from microorganisms, biosynthesized Bi_2_O_3_ NPs exhibit a reduced hazard profile due to the general use of ethanol and distilled water as solvents in producing plant extracts. Bi_2_O_3_ NPs derived from plant extracts are extracted from various tree parts, including the bark, roots, leaves, flowers, fruit extracts, and shells [144]. Separating the BiNPs-producing bacterial strain (named *Delftia* Spp. SFG) from salt marsh allowed for further purification, characterization, and elucidation of the biogenic BiNPs' cytotoxic and antioxidant properties [197]. An additional inquiry involved the utilization of a fruit peel extract of *Nephelium lappaceum L*. to produce Bi_2_O_3_ nanoflakes [198]. Currently, a one-step reduction method is being employed by researchers to produce biomolecule-mediated BiNPs. BiNPs were synthesized using an assortment of biomolecules, including bovine serum albumin, human serum albumin, and gelatin [199]. In dentistry and medicine, NMs have been utilized as novel, alternative antibacterial agents in recent years. Bi subsalicylate (BSS) has been employed as an antibacterial agent; however, its potential effectiveness against the bacteria responsible for periodontal disease in the form of NPs (BSS-nano) has not been exhaustively investigated. The objective of the researcher" 's study was to assess the safety profile of BSS-nano through an examination of its cytotoxicity in human gingival fibroblast (HGF-1) cells and its antibacterial effectiveness against oral anaerobic bacteria. It was demonstrated that BSS-nano has a principal dimension of 4–22 nm and a polygonal shape. Antibacterial agents may be incorporated into dental materials and antiseptic solutions via BSS-nano [200]. Studies on the antibacterial effects of zerovalent BiNPs are in their infancy but promising. Similar to Chlorhexidin (CHX), they were effective in preventing the spread of *S. mutans*. Consideration must be given to the fact that zero-valent BiNPs inhibit bacterial growth at a MIC of 0.5 mM before their incorporation into mouthwash. CHX, the industry standard for oral antiseptics, has been shown to exhibit comparable effects to these NPs in the conducted experiments. The introduction of zerovalent BiNPs halted *S. mutans* biofilm production entirely. This result was unexpected, as it was expected that zero-valent BiNPs would inhibit cell proliferation but not wholly halt it. It was postulated by the researchers that the inactivation of 69% of the cells by NPs would result in an insufficient number of cells remaining to generate a biofilm. Based on the preponderance of experimental evidence, these NPs appear to be a viable therapeutic option for biofilm-associated bacterial infections [143]. The utilization of GS in this NP has played a crucial role in advancing clinical applications for the management of patients with periodontitis.

Cobalt oxide NPs (Co_3_O_4_-Nps) have numerous applications, and ecological synthesis methods are currently favored over other approaches due to the benefits associated with their production. The present investigation involved the synthesis of Co_3_O_4_-Nps via the Safaida leaf extract of Populus ciliata and Co nitrate hexahydrate, which served as the Co source. The antibacterial properties of the synthesized Co_3_O_4_-Nps were assessed about gram-negative and gram-positive bacteria. The results indicated that the compounds exhibited activity against *E. coli*, *B. subtillus*, *B. lichenifermia*, and *K. pneumonia*. The one-way analysis of variance (ANOVA) was employed to statistically analyze the activity results in conjunction with "Dunnett's Multiple Comparison Test." The mean activity levels were found to be highest for *B. subtilis* (21.8 ± 0.7) and lowest for* E. coli* (14.0 ± 0.6) [201]. A newly synthesized low-dimensional Co(II) coordination complex was achieved by combining a semi-rigid ether-based unsymmetrical tetracarboxylic acid with the ancillary dipyridyl ligand 4,4′-bipyridine (bipy) using solvothermal reaction conditions. Concurrently assessing its efficacy in treating chronic periodontitis, the underlying mechanism was investigated. The findings from the real-time RT-PCR analysis further validated that the compound exhibited a dose-dependent ability to substantially suppress the relative expression levels of survival *genes in Porphyromonas gingivalis*. The compound shows promise as a viable candidate for treating chronic periodontitis through its ability to inhibit the expression of survival genes associated with *Porphyromonas gingivalis* [202].

## Future prospective

Historically, oral health issues have been treated with herbal remedies. It has been demonstrated that GS of Ag, Au, Zn, Cu, and Fe NPs enhanced with plant compounds is superior to conventional materials in treating numerous dental disorders. The use of nanostructures in periodontitis diseases is observed. NPs are utilized in the placement of dental implants, prostheses, and oral disease prevention medications. Pharmaceuticals and oral fluids can also be delivered via NMs, thereby facilitating treating oral malignancies and enhancing oral healthcare. In addition to toothpaste and mouthwash, these are present in additional dental care products. However, knowledge regarding the safety of NMs is limited, which calls for further research. Many issues, including drug resistance, could potentially be resolved with NPs manufactured by GS [136]. "Green" synthesis has garnered significant interest in the field of materials science due to its reputation as a dependable, environmentally favorable, and sustainable method for producing a diverse array of materials and NMs, such as bioinspired materials, metal/oxide NMs, and hybrid materials. As a result, GS is considered a crucial instrument for mitigating the harmful effects associated with conventional synthesis methods for NPs that are widely employed in industry and laboratories [203]. Recently, metal oxide NPs have come to be recognized for their significant commercial utility. Nonetheless, the potential toxicity of these NMs has been an additional considerable area of research interest. Consequently, the GS of these particles is a crucial solution for guaranteeing reduced toxicity levels and facilitating their unimpeded incorporation into consumer goods intended for human use. While it may seem simplistic, the utilization of microorganisms and plant extracts in the biological synthesis of metal oxide NPs presents tremendous potential for developing economical and environmentally friendly particles that may find utility in the healthcare industry. Furthermore, it is imperative to conduct toxicity assessments on NPs and establish robust risk management protocols for their synthesis, materials handling, storage, and disposal [204]. Herbal medicine has been used to treat oral and dental diseases and provide daily care since antiquity. Because biogenic metal NPs mediated by plants can circumvent the drawbacks of herbal remedies, incorporating herbal medication into NP formulations in dentistry represents an innovative breakthrough. It has been demonstrated that the ecological synthesis of metal NPs (Ag, Cu, and AuNPs) using medicinal plant extracts is more effective than conventional materials at treating various oral and dental diseases [205].

NPs can be produced chemically or via GS; however, the chemical synthesis of NPs is labor-intensive, costly, and energy-intensive. Due to their facile functionalization, AuNPs have the potential to serve as antimicrobial agent carriers. Nevertheless, implementing physical or chemical processes to generate NPs is prohibitively expensive, labor-intensive, and detrimental to ecosystems. Conversely, the GS of NPs is more economical and consumes less energy [206]. The harmful side effects of organic solvents and the toxicity of reducing reagents used in the chemical production of AuNPs prompted the search for environmentally friendly alternatives. AuNPs, a type of NMs, are readily produced via a one-step green chemistry procedure that is favorable to the environment. Their non-toxicity and biocompatibility are renowned worldwide. AuNPs are up-and-coming candidates for biological applications due to their unique properties. The diagnostic utility of AuNPs in periodontal disease is substantial owing to their distinctive and crucial optical characteristics [145, 146]. Dental caries and periodontal disease-causing microorganisms, including *S. mutans*, *S. aureus*, *L. acidophilus*, *M. luteus*, *B. subtilis*, *E. coli*, *P. aeruginosa*, *C. albicans*, were used to assess the antimicrobial properties of green synthesized AgNPs and drug-blended AgNPs. The AgNPs, in combination with drug-blended AgNPs, exhibit noteworthy antibacterial and antifungal properties. The MIC values of AgNPs, as determined against specific microorganisms that cause dental caries and periodontal disease, are observable within the concentration range of 25–75 μg/mL [163]. The GS of iron NPs is becoming increasingly prevalent as a cost-effective and environmentally sustainable therapeutic approach. The antimicrobial effects of biologically generated IONPs were evaluated using Gram-negative and Gram-positive microorganisms. The antibacterial activity of IONPs was demonstrated by the fact that they inhibited bacterial strains within a significant zone. Through the synergy of IONP, the antibacterial activity of both natural and synthetic medications is enhanced. The efficacy of prevalent natural substances, such as green tea leaves, neem buds, and clove buds, against *S. mutans* was investigated. To assess the impact of different treatment combinations on *S. mutans*, investigators utilized three others plant extracts: one alone, one in combination with IONP, and one with both IONP and amoxicillin. The efficacy of an antimicrobial agent is augmented when it is combined with IONP [207, 208]. ZnO NPs have garnered significant attention in antibacterial research [209]. ZnO has been proposed as a potential material for dental fillings due to its antimicrobial properties. Consequently, practicable administration lubricants containing ZnO were formulated to treat periodontitis [210]. Green TiO_2_ NPs exhibited encouraging antimicrobial and antifungal properties against various microorganisms. Environmentally produced TiO_2_ NPs exhibit remarkable antibacterial (*S. mutans*) and antibiofilm properties [183, 211]. The utilization of Cu and Cu-based NPs, composed of inexpensive and abundant Cu metal, has garnered significant attention in recent times [212]. The utilization of numerous plant components or entire plants for the GS of CuNPs is attributable to the abundance of bioactive compounds found in plants. Synthesized CuNPs have been derived from preparations of plants discovered in various parts of the world. Multiple CuNP formulations have been utilized by scientists in periodontal therapy [213].

It is crucial to understand that the emergence of NPs is accompanied by safety, environmental and ethical problems, despite their enormous promise. To guarantee their appropriate and sustainable deployment, comprehensive research and regulatory monitoring are required in light of concerns about their long-term effects on human health and ecosystems [214]. Any dental or medical nanoproduct that has completed its research and development phase is subjected to a rigorous preclinical in vitro testing protocol to examine its mechanical, immunological, and toxicological qualities. Guidelines for reviewing the dangers of NMs have been established by several organizations, including the National Institute of Occupational Safety and Health and the US Environmental Protection Agency. Legislative challenges are persistent when creating a multidisciplinary regulatory framework to evaluate and manage nanotechnology and address ethical issues that fall into the four areas of metaphysics, equality, privacy, and security. As a result, it is imperative that participants comprehend the degree of danger linked to their exposure to novel substances and information and that each clinical trial establish safety monitoring boards tasked with diligently monitoring and documenting any early-onset adverse effects, identifying irregularities in data management, and safeguarding the welfare and security of test subjects. The inherent unpredictability of NMs presents dentists with an ethical quandary when confronted with an extensive selection of materials, some of which have established clinical utility through short-term clinical studies (e.g., nano filled composite resins). In contrast, others, including hybrid or micro filled composite resins, are conceptually appealing but lack such support. The conventional method of making ethical decisions, which primarily relies on utilitarianism, is not up to speed with the rapid advancements in nanotechnology and its unpredictable future. Because of this, a deeper comprehension of the science is needed, along with risk/benefit assessments and ethical concerns at every stage of development. This resulted in the concept of anticipatory ethics and governance, created to use ethical analysis models to identify and address ethical and societal implications when the technology is still in its early stages. This allows for easy modification and guidance of the technology towards an ethically acceptable outcome [215, 216]. Consequently, the decision to utilize NMs is contingent upon the specific clinical situation and the tooth that requires restoration, with careful consideration given to aesthetic requirements, loading, and potential hazards like parafunctional habits. Ongoing research aims to enhance the performance of current NMs. Possible future developments include nano-biosensing devices that are more cost-effective and efficient, capable of accurately diagnosing oral cancer. Additionally, novel oral drug delivery systems are being developed to impede biofilm formation and decrease the prevalence of periodontal disease and dental caries. While the scientific principles underlying nanotechnology are captivating, their extensive clinical application is limited by the absence of long-term clinical evidence about their efficacy [215].

Plant NPs have the potential to be utilized more extensively in the prevention of oral diseases, treatment of oral cancer and prostheses and implants, and oral health care. While there have been numerous demonstrated benefits of NPs, they also possess specific disadvantages, including exorbitant expenses, the potential for respiratory disease through simple inhalation, and alterations in homeostasis. A novel subfield of toxicology, nanotoxicity investigates the adverse effects of NPs, which may have toxicological ramifications. The NPs are incredibly reactive and induce numerous adverse molecular effects due to their minute size. Although most plant extract NPs are unprocessed, they are not the preferred functional molecules for plant extracts. To produce NPs at a minimal cost, every active group of plant extracts is examined. Determining which molecule functions as an RA or stabilizer agent (SA) and identifying the biological NPs responsible for therapeutic applications are highly complex. Primarily, the in vivo evaluation of the system's overall toxicity should be pursued. Acquiring the recommended dosage of compensatory doses for NMs produced environmentally will prove to be a formidable task. Future research is necessary to determine the long-term effects of green NPs on numerous clinicians [136]. Notably, GS-mediated NP synthesis is an efficient, economical, and rapid method that has enabled nanotechnologists to fabricate desirable NMs via renewable energy processes. This technique is economical due to the absence of a requirement for an external stabilizing agent. Biological synthesis has several notable advantages over physical and chemical methods. Firstly, it is a clean and environmentally beneficial process, utilizing benign compounds. Secondly, it makes use of renewable sources. Lastly, active biological components such as enzymes and phytochemicals contribute to the reduction process [217, 218].

Their application in the clinic, however, is entirely restricted. To ensure their safe application, these NPs must undergo exhaustive testing for adverse effects. In addition, when implementing the circular economy concept, it is necessary to consider the availability of primary materials and their processing costs, as well as their recyclability and sustainability post-use. As for treating periodontitis, the primary antibacterial mechanism by which different metal NPs exert their effects is unknown. Research conducted in vivo is essential for precisely assessing the medicinal potential of metal NPs and determining how microbes react to these factors. To fully understand their role in biological systems, in-vivo investigations are essential. Furthermore, data from the publications reviewed here indicates that the investigation into using metal NPs to treat periodontitis is still in its early phases. A significant portion of the studies examined in this article are technical and frequently do not include a principal expense/benefit analysis or detailed descriptions of the fundamental procedures involved in each investigation. The mechanisms underlying the cellular uptake of metal NPs and their antibacterial properties in treating periodontitis require additional research. It is important to note that most site-specific transfer mechanisms perform admirably in vitro but inadequately in vivo. Thus, research for periodontitis therapy in vivo could benefit this investigation [31]. Although living sustainably is a desirable goal, there are several potential drawbacks to green technology and processes, including high implementation costs, ignorance, and the absence of substitute chemicals or raw materials. Although there are many benefits to green NMs synthesis, there are drawbacks. These include problems with raw material selection, reaction conditions, product quality control, and application. These elements challenge the creation of environmentally friendly NMs for production and widespread use. Researchers found that various easily found plants in the area may be used to make green NPs. These studies indicate that although full use of native plants is feasible, significant global NMs production remains challenging. Utilizing raw components in actual manufacturing may be difficult due to time constraints. Throughout the blossoming stage, the cotton leaf should gather the ingredients required to make Ag NPs [64, 219, 220]. Furthermore, the ideal temperature for several environmentally friendly synthetic processes is high, and the synthesis process takes a long time, requiring a significant amount of energy that might be harmful to the environment. Even with ecologically friendly starting materials, the procedure doesn’t always follow sustainable synthesis guidelines. The NPs generated by distinct extracts exhibit significant variation in form and size, with the measured quality being inadequate. Current sources state that because of the wide variations in particle diameter, green technology is inappropriate for large-scale production, and managing particle size becomes a significant difficulty throughout the manufacturing process. Only a recent study was able to show how plant extracts affected synthesis, and even then, the precise molecular processes involved remain mysterious. The NPs produced by different quotes differ significantly in size and shape, and the properties found are insufficient. Current sources indicate significant differences in particle size, which renders green technology inappropriate for large-scale production and presents substantial challenges for controlling particle size throughout manufacture [221–223]. In addition, several challenges, such as poor yield, irregular particle size, complex separation procedures, periodic, local raw material accessibility, and much more, need to be overcome before sustainable NMs synthesis and its uses can be achieved. There are now many different green NM synthesis techniques and processes accessible, and more will be developed in the future [223].

## Comparison of nanoparticles with each other

AuNPs have unique qualities that make them useful in dentistry. They may be used innovatively for dental caries, bone regeneration, periodontology, implantology, tissue engineering, and cancer diagnostics. It may be used as an addition to different dental materials because of its antimicrobial and antifungal properties. The killing power of *S. mutans’* lower temperature plasma is increased by AuNPs. By adding AuNPs, the antibacterial activity of the material may be increased, and the likelihood of secondary caries can be decreased. According to in-vitro studies, AuNPs have a role in periodontal regeneration because they stimulate the growth of periodontal ligament cells with particles as small as 60 nm. They have also shown the ability to block the production of biofilms, making them a valuable tool for periodontology preventative therapy [148, 224].

It could be possible to produce AgNPs in an affordable, sustainable, and environmentally friendly manner using techniques that eliminate the plant material. Plants provide a better, safer, and more affordable option for physical and chemical procedures because of their phytochemical components. Given that AgNP's toxicity and efficacy are dependent on both size and shape, synthesis techniques and protocols have garnered a great deal of attention in the scientific community recently. AgNPs may be produced chemically, biologically, or physically. While the chemical process is risky and expensive, physical procedures need a lot of energy to sustain the high pressure and temperature required for the reaction. Because of the drawbacks of NMs, such as their toxicity to bone cells, variable biocompatibility based on size, surface, and composition, and high cost, biochemical techniques or biosynthesis have been developed, such as the use of plant-based biomolecular extracts [225]. Before using NPs in the biomedical area, it is crucial to use biocompatible materials. This work showed for the first time the antibacterial activity of AgNPs stabilized with the biocompatible tripeptide glutathione against typical periodontal microorganisms. Against *S. mutans*, the NPs demonstrated potent antibacterial properties. Low NP concentrations (6.16 µg/mL) did not significantly affect cell viability during the GSH-AgNP cytotoxicity assays in HGF-1 cells, as cell viability remained over 90%. At doses of 24.63 µg/mL, however, viability was lost by more than 40%. Ag quantity stays in the HGF-1 cells, and the cellular absorption of Ag (1–4%) exhibited an inverse relationship with the initial GSH-AgNP dosage. This was in line with the NPs' concentration-dependent toxicity. When GSH-AgNPs were present, fibroblasts produced ten times more IL-8 (or TNF-α) than IL-6 or other immune markers. An unfavorable association was found between the generation of cytokines and cell viability at GSH-AgNPs concentrations (≥ 12.31 μg/mL Ag concentration) that impair cell viability. The investigation results add to the knowledge of the impact of AgNPs in the oral cavity. It also raises additional concerns about using this NMs in periodontal and other dental applications for antibacterial therapies. Specifically, the concentration of AgNPs must be optimized in proportion to their cytotoxic, inflammatory, and bacterial effects [226]. AgNPs showed more antibacterial activity (80–100%) against both Gram-positive and Gram-negative bacteria as well as yeast than AuNPs (0–87.92%), suggesting that they might be used as antimicrobial agents [227].

TiO_2_ NPs are extensively used in the food sector and are often found as ingredients in a range of medicinal and cosmetic items, including toothpaste and sunscreens. Even with the extensive use of TiO_2_ NPs, little is known about their biological impacts and the underlying processes of cellular response. As such, it is critical to have a comprehensive understanding of the toxicological properties of this material. TiO_2_ NPs can potentially be hazardous, and the main reason for this appears to be the production of ROS. These ROS may induce oxidative stress, inflammation, genotoxicity, metabolic changes, and even cancer. Some chemical and physical characteristics of TiO_2_ NPs that significantly affect the kind and degree of cell damage include their size, crystal structure, and photo-activation. To ultimately use it in treating periodontitis, further clinical research and investigations are needed [228].

According to recent research, using hybrid metal NPs in combination may increase their bactericidal efficacy [229]. Antibiotic NP hybridization might thus be a novel tactical method for combating these pathogenic microorganisms. Noble metals, like Ag NPs, may become more biologically active due to faster ionization resulting from hybridization with nano-Fe_3_O_4_ (or nano-MnO_2_). However, the hybridization of semiconductor oxides (ZnO or TiO_2_) with noble metals (Ag or Au) also increased the production of ROS [230]. For instance, the purpose of this study was to highlight the potential clinical benefit of Ag-doped TiO_2_ nanotubes for providing antimicrobial properties against the adhesion of peri-implantitis-associated bacteria *A. actinomycetemcomitans*, *Tannerella forsythia*, and *Campylobacter rectus* for transmucosal components of dental implants. Ag content in TiO_2_ nanotubes increased dramatically during annealing. When tested against Ag-doped TiO_2_ nanotubes as-annealed against *A. actinomycetemcomitans*, *T. forsythia*, and *C. rectus*, they demonstrated antibacterial solid activity [231]. The most widely used ZnO NPs have a few advantages over AgNPs, including being less expensive and having a whiter appearance. Proteins are bonded to one another by toxic heavy metals. The thiol groups of vital enzymes are strongly related to heavier metals, effectively rendering them inactive. It is believed that once metal NPs like Ag bind to a protein's functional groups, the protein becomes denatured and inactive. It was shown that in intact heterotrophic biofilms, exposure to UV light and surface-functionalized TiO_2_ NPs did not affect alkaline phosphatase (ALP) activity. However, *E. Coli'*s secreted ALP enzyme is severely inhibited at concentrations of ZnO NPs much lower than those seen in full biofilms [232, 233]. A local antibacterial system based on micro sized multifunctional Ag-TiO_2_-x encapsulated in ATA microspheres has been reported. Researchers discovered that ATA showed moderate photocatalytic activity and strong photothermally enhanced dual enzyme-mimicking (peroxidase-like and catalase-like) activities in low H_2_O_2_ concentrations. As a result, the ATA/H2O2/NIR combination demonstrated vigorous antibacterial activity against *P. gingivalis* and *S. gordonii* in both their planktonic and biofilm forms. Ag^+^ may be released by ATA with the help of ROS in sufficient amounts to make it effective against periodontal bacteria. Furthermore, it was expected that the oxygen produced locally would enhance the hypoxic environment and reduce the inflammatory reaction of periodontal stem cells to lipopolysaccharide. In an in vivo rat model of periodontitis, the ATA/H_2_O_2_/NIR combination reduced the bacterial load, reduced inflammation, and encouraged tissue healing. They developed a unique method for oxygenating periodontitis and providing targeted, long-lasting antibacterial treatment. This approach does not employ antibiotics or NMs, and it has shown great potential in various biological applications for supplementary periodontitis treatment [234, 235].

It has been shown that CuONPs are antibacterial and inhibit the growth of biofilms. The large surface area/volume ratio of Cu NPs enhances their antibacterial action. The exact mechanism by which CuNPs destroy microbes is still unknown after much investigation. CuNPs have better bactericidal activity than AgNPs, a different type of NP frequently used in biomedical research against *E. Coli*, *B. subtilis*, and *S. aureus*. CuNPs have antimicrobial and metallic properties that make them attractive for dental applications. These NPs composites are easily made using common dentistry supplies and are said to be physiochemically stable. Their use in the clinic is, however, minimal. CuNPs have been the focus of most dental research since they are an antibacterial agent and an amalgam modifier. CuNPs, the focus of the current study, are said to be beneficial when added to dental cement, restorative materials, adhesives, resins, irrigating solutions, obturations, orthodontic archwires and brackets, implant surface coatings, and the bone regeneration process. Compared to AgNPs, CuONPs are less costly, environmentally safe, and chemically stable. CuONPs are less expensive, more ecologically friendly, and have a more stable chemical structure than AgNPs [31].

According to some research, gram-negative bacteria may have an enrichment of Fe_3_O_4_ particles between their inner and outer membranes while acting as nanozymes. This can result in a more potent antibacterial impact than gram-positive bacteria. Fe_3_O_4_ particles show promise when used in the treatment of periodontitis since gram-negative bacteria are the predominant bacteria in the condition. Since Fe_3_O_4_ NPs do not pose a substantial risk to animals, they are widely used to touch human tissues [236, 237]. Here, a layer-by-layer approach was used to create a particular kind of antibacterial magnetic nanoparticles (AMPs), which were shown to be a helpful substitute for the focused treatment of periodontitis. AMPs demonstrated exemplary performance in bactericidal effect, reproducible recovery, and cytocompatibility, as shown by the cyclic antibacterial performance, cytotoxicity, and bactericidal rate. The research of antibacterial processes indicates that AMPs cause bacterial lysis and death by rupturing bacterial cell membranes in addition to inhibiting the action of bacterial respiratory chain dehydrogenase. Furthermore, about 80% of the bacterial biofilms that have developed may be eliminated. More crucially, an in vivo investigation showed that using AMPs to treat periodontitis following the formation of alveolar bone might dramatically reduce the condition or perhaps cure it. In summary, researchers establish a basis for the creation of AMPs as periodontal pocket medication carriers for the management or avoidance of periodontitis [238].

There are several potential medical applications for metal-phenolic networks (MPNs), which are nanoplatforms of multifunctional hybrid NMs created by coordinating metal ions with polyphenols. As a result, MPNs were applied to the surface of AuAg@PC-Fe, or branching AuAg NPs. Procyanidin (PC)-Fe network relieved oxidative stress and excessive inflammation and improved the photothermal characteristics of AuAg NPs to produce efficient photothermal antibacterial activity against periodontal infections. By stimulating the phosphoinositide 3-kinase/protein kinase B signaling pathway and upregulating nuclear factor erythroid 2-related factor 2, AuAg@PC-Fe helps polarize alternatively activated macrophages. This is achieved by scavenging ROS and suppressing the nuclear factor kappa-B signaling pathway, which regulates immunity. In vivo, repair capacity of periodontal inflammatory tissue was enhanced. This design offers novel approaches to using MPNs in immunotherapy and photothermal treatment. It provides a fresh approach to treating infectious disorders, including periodontitis [239].

## Conclusion

Metal NPs are highly promising due to their potent antibacterial activity. Because metallic NPs inhibit the proliferation of numerous microorganisms, they are pertinent to treating periodontitis. Hence, the innovative metal NPs offered a distinct perspective on advancing productive antibacterial and anti-inflammatory frameworks for the management of periodontitis. Nevertheless, different methodologies may be necessary for laboratory synthesis on a limited scale and industrial-scale production. Synthesis of complex structures, synthesis of nanodispersed particles, size and shape control, and reproducibility are some of the most significant obstacles in synthesizing NMs. In addition to plants, metal NPs have been synthesized utilizing a variety of microorganisms, including bacteria, fungi, and yeast. In recent decades, GS of NMs has emerged as an environmentally benign and sustainable technology for removing pigments. Particularly, leaf extracts from plants have been regarded as functional and cost-effective substances for the synthesis of NPs. Numerous researchers have utilized the GS method to prepare metal/metal oxide NPs from plant leaf extracts to investigate their diverse applications. Biomolecules found in plants, such as carbohydrates, proteins, and coenzymes, have remarkable potential for reducing metal salts into NPs. GS is superior to conventional chemical synthesis in terms of cost-effectiveness, pollution reduction, and enhancement of environmental and human health safety. Furthermore, the elimination of hazardous compounds that are known to cause toxicity enhances the biocompatibility of the final product with healthy tissues, thereby improving its suitability for in vivo applications. Hence, considering the recent advancements and continuous endeavors to enhance the GS of metal NPs and investigate their potential biomedical uses, it is optimistic that the implementation of our methodology on a significant scale, as well as their commercial implementation in treating periodontitis, will prove to be exceedingly beneficial in the coming years.

## Data Availability

Not applicable.
